# Experimental Manipulation of Pectin Architecture in the Cell Wall of the Unicellular Charophyte, *Penium Margaritaceum*

**DOI:** 10.3389/fpls.2020.01032

**Published:** 2020-07-08

**Authors:** Kattia Palacio-Lopez, Li Sun, Reagan Reed, Eric Kang, Iben Sørensen, Jocelyn K. C. Rose, David S. Domozych

**Affiliations:** ^1^ Department of Biology, Skidmore College, Saratoga Springs, NY, United States; ^2^ Plant Biology Section, School of Integrative Plant Science, Cornell University, Ithaca, NY, United States

**Keywords:** pectins, cell wall, *penium*, rhamnogalacturonan I, homogalacturonan lattice, abiotic stress

## Abstract

Pectins represent one of the main components of the plant primary cell wall. These polymers have critical roles in cell expansion, cell-cell adhesion and response to biotic stress. We present a comprehensive screening of pectin architecture of the unicellular streptophyte, *Penium margaritaceum*. *Penium* possesses a distinct cell wall whose outer layer consists of a lattice of pectin-rich fibers and projections. In this study, cells were exposed to a variety of physical, chemical and enzymatic treatments that directly affect the cell wall, especially the pectin lattice. Correlative analyses of pectin lattice perturbation using field emission scanning electron microscopy, confocal laser scanning microscopy, and transmission electron microscopy demonstrate that pectin lattice microarchitecture is both highly sensitive and malleable.

## Introduction

Pectins constitute a diverse group of galacturonic acid (GalA)-containing polysaccharides that form a dynamic structural matrix in the primary cell wall of plants. Pectins are integral to the maintenance of wall infrastructure, wall porosity, cell-cell adhesion and defense against pathogens, as well as being key polymers in the regulation of primary wall expansion (Willats et al., 2001; Mohnen, 2008; Caffall and Mohnen, 2009; Agoda-Tandjawa et al., 2012; Cosgrove, 2014; Yapo and Gnakri, 2015; Anderson, 2016; Voxeur and Höfte, 2016). Pectins may account for up to 35% of the polysaccharide constituents of the primary cell wall and are categorized into several classes, including homogalacturonan (HG), rhamnogalacturonan I (RGI), and rhamnogalacturonan II (RGII), apiogalacturonan, and xylogalacturonan (Mohnen, 2008). HG and RGI represent the most abundant forms found in the primary cell wall. HG can constitute up to two thirds of the primary wall pectin and consists of a linear polymer of (1-4)-α-D-GalA that can be methyl esterified at the *C*-6 carboxyl group. Methylesterified-HG chains can be de-methylated following secretion to the wall by pectin methylesterase (PME) enzymes and the resulting carboxyl groups, if present on adjacent HG chains, can coordinate calcium ions (Ca^2+^) and form interchain cross-links (Phyo et al., 2017). This results in a physical transformation of soluble HG to a stiff gel, which increases wall rigidity (Peaucelle et al., 2011; Palin and Geitmann, 2012). Recent work (Haas et al., 2020) has also elegantly demonstrated that HG forms nanofilaments that structurally change based on methylation level. RGI, which constitutes up to a third of cell wall dry weight, is a heteropolymer with a backbone of a repeated [-α-d-GalA-1,2-α-l-Rha-1,4-]_n_ disaccharide, decorated with complex side chains of 1,5-linked arabinans, 1,4-linked galactans, or type I and II arabinogalactans (Mohnen, 2008; Caffall and Mohnen, 2009; Yapo and Gnakri, 2015). RGI glycan structure is quite variable and has been proposed to act as a scaffold for other pectic polysaccharides that form a matrix or domain (Vincken et al., 2003) that is essential for cell wall extensibility and firmness. RGI contributes to the distribution of the mechanical loads on the network of cellulose microfibrils that ultimately control cell wall expansion under conditions of high turgor pressure (Bidhendi and Geitmann, 2016).

Recently, the study of pectins *in muro* has benefited significantly from the application of analyses of mutants associated with pectin biosynthesis (Francocci et al., 2013; Biswal et al., 2018; Wang et al., 2019), high resolution microscopy using pectin-specific probes (Ralet et al., 2010; Anderson et al., 2012; Mravec et al., 2014; Mravec et al., 2017a; Guo et al., 2019; Zhao et al., 2019), super resolution three-dimensional direct stochastic optical reconstruction microscopy (3D-dSTORM; Haas et al., 2020), atomic force microscopy (Kirby et al., 2008; Paniagua et al., 2014; Imaizumi et al., 2017), and solid state nuclear magnetic resonance spectroscopy (Wang et al., 2015). These studies have shown that the microarchitecture of pectic polysaccharides in the wall is highly complex and modulates during cell expansion, development and in response to external abiotic and biotic stress. For example, it has been shown that pectin backbones have both mobile and rigid domains that are both positioned between cellulose microfibrils and structurally interacting with cellulose (Phyo et al., 2017). Changes to these domains directly affect microfibril mobility and wall/cell expansion and morphogenesis. However, major challenges remain in the quest to elucidate pectin structure, dynamics, and interpolymeric interactions. This is due to the innate structural complexity of plant cells walls that limit our ability to resolve specific polymers in the dynamic wall infrastructure. For example, in multicellular plants, it is exceptionally difficult to resolve fine structural features or secretion mechanisms of specific wall polymers in an individual cell that is surrounded by, and interacting with, other cells within a tissue/organ.

Over the past decade, basal streptophytes or Charophycean Green Algae, i.e., the group of extant green algae that are most closely related and ancestral to land plants (Delwiche and Cooper, 2015; Rensing, 2018), have been shown to contain many of the cell wall polymers found in land plants (Sørensen et al., 2011). Pectins are often major constituents of basal streptophyte walls. They are products of complex biosynthetic pathways (Boyer, 2009; Jiao et al., 2020) and often display distinct modes of post-secretion incorporation into the wall architecture (Proseus and Boyer, 2012a; Proseus and Boyer, 2012b; Eder and Lutz-Meindl, 2010; Domozych et al., 2014a). Furthermore, basal streptophytes’ relative small sizes, simple morphology, and ease in culturing/experimentation make them outstanding specimens for cell wall studies (Domozych et al., 2016). *Penium margaritaceum* is a unicellular streptophyte (Zygnematophyceae) that produces a unique cell wall that is highlighted by an outer pectic layer of highly structured, Ca^2+^-complexed HG, referred to as the “lattice” (Domozych et al., 2014a). This layer is connected to an inner cellulosic layer *via* an embedded medial layer containing RGI. The *Penium* HG lattice can be conveniently labeled with monoclonal antibodies (mAbs) and other probes in live cells and subsequent pectin deposition patterns may be directly monitored using fluorescence microscopy. The fast growth rate and unicellular phenotype of *Penium* also allow for rapid experimental interrogation with various stress-inducing agents.

In this study, we report on a comprehensive structural and experimental screening of pectin architecture using field emission scanning electron microscopy (FESEM), confocal laser scanning microscopy (CLSM), and transmission electron microscopy (TEM). We demonstrate that the pectin architecture is highly malleable when cells are interrogated with chemical, enzymatic, and physical stress agents.

## Materials and Methods

### Algal Growth Conditions

*Penium margaritaceum* Brébisson (Skidmore College Algal Culture Collection, clone Skd#8) was maintained in sterile liquid cultures of Woods Hole Medium (Nichols, 1987) supplemented with soil extract (WHS), pH 7.2 at 18 ± 3°C in a photoperiod of 16 h light/8 h dark cycle with 74 µmol photons m^-2^ sec^-1^ of cool white fluorescent light. For some of the enzyme treatments, the pH of the medium was adjusted to 6.0, 7.2, or 8.0 in order to match the optimal pH of the externally applied enzyme (see below), as specified by the supplier. For media that provided different Ca^2+^ levels or addition of Ba^2+^, WHM (without soil extract), or CaCl_2_ was used as the base. Cells were subcultured every week and cells from log-phase culture (7 to 14 days old cultures) were used for the experiments.

### Monoclonal Antibodies and Probes

JIM5 (specificity: Homogalacturonan, HG, with low degree of methyl-esterification; Clausen et al., 2003) and JIM7 (specificity: Homogalacturonan, HG, with high degree of methyl- esterification; Clausen et al., 2003) were obtained from Plant Probes, Leeds, UK (http://www.plantprobes.net). CBM64a-GFP (specificity: A carbohydrate binding module derived from *Spirochaeta thermophile* that binds to crystalline forms of cellulose; NZYtech, CZ0499; https://www.nzytech.com/). INRA-RU1 (specificity: Rha-(1,4)-GalA-(1,2)-Rha-(1,4)-GalA-(1,2)-Rha-(1,4)-Rha-(1,4)-GalA-(1,2)-Rha-(1,4)-GalA-(1,2)-Rha-(1; Ralet et al., 2010) was provided by Dr. Marie-Christine Ralet (INRA, Nantes, France). OGA7-13^488^ binds HG with a low degree of esterification, based on the complexation with calcium and the details of the specificity towards HG with different degree of esterification can be found in Mravec et al., 2017b.

### Experimental Treatments

Cells were collected by centrifugation (800 × g, 1 min) and pellets were resuspended in fresh growth medium (either WHS or WHM). The cells were vigorously shaken and then re-centrifuged as above. This was repeated three times. This represented the washing protocol that removed extracellular polymeric substance (EPS) from the cell surfaces. Experimental studies were performed in 1 ml volumes of WHS plus experimental agent containing 2,000 cells/ml +/-200 cells in triplicate wells of 12-welled microplates (Fisher Scientific, Pittsburgh, PA, USA). Experimental conditions included treatment with 225 mM sorbitol, 8 µg/ml cytochalasin B (CB; Sigma Chemical), 1 µM amiprophos-methyl (APM; Sigma Chemical), or µM epigallocatechin (Sigma Chemical). For EDTA extraction treatments, cells were treated in 50 mM EDTA for 90 min (full extraction) or 10 min (partial extraction) in 0.01 M MES (pH 6.0). For pectin-targeted enzyme treatments, cells were treated with endo-1,5-α-l-arabinanase from *Aspergillus niger* (# E-EARAB, 0.6U; Megazyme, Bray, Ireland; pH 6.0), α-l-rhamnosidase (# E-RHAMS, 4.5U; Megazyme; pH 6.0), rhamnogalacturonan lyase from *Cellvibrio japonicus* (# CjRgI11A, NZYTech, Lisbon, Portugal; 3 µl/ml; pH 7.2), rhamnogalacturonan hydrolase (RGIase, 3 μl/ml; courtesy of Dr. Marie-Christine Ralet; pH 7.2), exo-polygalacturonase (exoPG) from *Yersinia enterocolitica* (# E-EXPGA, 9U; Megazyme; pH 6.0), endo-polygalacturonase (endoPG) from *Aspergillus aculeatus* (# E-PGALUSP, 65 U; Megazyme; pH 7.2), plant PME (orange peel PME; Sigma: P5400, pH 8.0; 1 unit/per mg protein; 1 unit releases 1 microequivalent of acid from pectin per minute), bacterial PME (5 µl/ml; from *Dickeya dadantii*; # CZ0791, NYZTech; pH 8.0; protein purity= 90%), pectin acetyl esterase (5 µl/ml; from *Dickeya chrysthamemi*; NZYTech #C20814; pH 7.2), or cellulase from *Trichoderma reesei* ATCC 26921 (200 µg/ml pH 8; Sigma). For controls, 1 ml samples of enzyme solutions were placed in boiling water for 5 min to denature, cooled and then dialyzed against 3 L of WHM (Sigma dialysis tubing, cut-off 6,000).

### Recovery Experiments

Cells were collected after the experimental treatments by centrifugation at 800 × g for 1 min. The supernatant was discarded and fresh WHS was added, followed by 10 s vortexing before a second centrifugation and supernatant discard. This washing procedure was repeated three times. Cells were resuspended in 1 ml of WHS and transferred to the well of a 12-multiwelled microplate. After 24, 48, 72, and 96 h, aliquots of cells were collected, and recovery was monitored after labeling with OGA7-13^488^ or Field Emission Scanning Electron Microscopy (FESEM).

### Live-Cell Immunolabeling

Live cell labeling was performed at room temperature and in the dark, and with constant gentle rotation. Cells were removed from experimental cultures, washed as above and incubated for 90 min in 1:10 (v/v) diluted hybridoma supernatant of the primary mAb solution in WHS. The cells were washed 3X with WHS, incubated for 90 min with 1:50 (v/v) diluted goat anti-rat (for JIM5, JIM7) or anti-mouse (for INRA-RU1) IgG conjugated with tetramethylrhodamine (TRITC) (Life Technologies™ Molecular Probes^®^, Eugene, OR, USA) secondary antibody in WHS. The cells were washed three times with WHS before microscopy imaging.

### OG7-13^488^ Labeling

Cells were collected by centrifugation, washed and incubated at room temperature in the dark with a 1:1,000 (v/v) dilution of OGA7-13^488^ in WHS with MES (Sigma Chemical) supplemented with 1 mM CaCl_2_ and adjusted to pH 5.8 for 90 min. The cells were washed 3X with WHS before microscopy imaging and washed 3X with WHS.

### CBM64A-GFP Labeling

Cells were washed 3x with WHS as described above and then fixed for 20 min at RT (room temperature) in a solution of 0.5% paraformaldehyde in WHS. The cells were then washed 3x in WHS and then incubated for 1 h at RT in a solution of WHS containing and 8U of pectate lyase (Megazyme). The cells were washed 3x in WHS and then incubated for 90 min at RT in the dark in a solution containing 5 µg/ml of CBM64a-GFP in WHS containing 0.05% Triton-X100. The cells were then washed 3x with WHS and viewed as described above.

### Light and Confocal Laser Scanning Microscopy

For routine-, differential interference contrast light microscopy (DIC-LM) and fluorescence microscopy (FLM), cells were viewed with an Olympus BX60 (Olympus America Inc., Melville, NY, USA) equipped with an Olympus DP73 digital microscope camera, using DP Controller 3.2.1.276 software. Confocal laser scanning microscopy (CLSM) was performed with an Olympus FluoView™ 300 or 1200 Confocal Microscope using Fluoview 5.0 with O3D software.

### Field Emission Scanning Electron Microscopy (FESEM)

Cells were collected, washed with WHS or WHM and placed in 0.05% Triton-X in WHS or WHM at 4°C for 10 min. The cells were then freeze shattered (Wasteneys et al., 1997), washed extensively with deionized water, frozen in liquid nitrogen and freeze dried. The dried cells were then brushed onto double-sided sticky tape (Ted Pella Inc.) on a Cambridge stub (EMS, Ft. Washington, PA) and sputter coated with gold-palladium (25 sec). FESEM imaging was performed using a ThermoFisher Quattra FESEM equipped with an UltraDry Premium EDS detector with full quantitative composition analysis. High resolution FESEM imaging involved a secondary electron detector with a 5 KV accelerating voltage, 2.5 spot size, and a 10 mm working distance under high vacuum conditions. The EDS (Energy Dispersive X-ray Spectroscopy) spectrum collection involved a 10 KV accelerating voltage, 5 spot size, and a 10 mm working distance (each spectrum was acquired over 1 min). We used the standardless quantitative analysis mode. The base spectrum and comparing spectrums were normalized by selecting a same energy range within the spectrum background.

### Transmission Electron Microscopy (TEM) and Immunogold Labeling

Sample preparation (including spray freezing and freeze substitution) for TEM was performed as described previously (Domozych et al., 2007). Thin sections (70 nm) of osmicated *Penium* in Spurrs resin (EMS) were cut with a Leica EM UC6 ultramicrotome (Leica Microsystems Inc., Bannockburn, IL, USA) with a diamond knife (DiATOME, Hatfield, PA, USA) and collected on Formvar-coated (EMS, Hatfield, PA, USA) copper grids. The sections were stained with conventional uranyl acetate/lead citrate and imaged with the TEM. For immunogold labeling, 60 nm sections were collected from non-osmicated cells embedded in London Resin (EMS) and deposited onto Formvar-coated nickel grids. These sections were treated for 2 min with 5% (v/v) hydrogen peroxide, washed with deionized water, treated for 10 min with 0.25% (w/v) ammonium chloride, rewashed, and blocked with 1% (w/v) blotting grade blocker (Bio-Rad) in phosphate buffered saline with 5% (v/v) Tween 20 (PBST) for 30 min at RT. Sections were washed with deionized water and incubated at 33°C for 90 min with a 1:10 dilution (in PBST) of the primary mAb. Sections were rewashed and re-blocked for 30 min, washed and incubated for 90 min at 33°C with a 1:50 dilution (in PBST) of goat anti-rat or anti-mouse IgG secondary antibody conjugated with 10 or 15 nm gold particles, for JIM5 and INRA-RU1 (Ted Pella, Inc., Redding, CA, USA). After a final wash with deionized water, both copper and nickel grids were stained with conventional uranyl acetate/lead citrate as above and imaged on a Zeiss Libra 120 kV TEM. For dual labeling, sections were labeled with JIM5-15 nm gold, followed by INRA-RU1-10 nm gold.

## Results

### General Experimental Considerations

In order to assure proper control and experimental consistency, we followed a strict protocol for the application/analysis of each experimental agent. First, we applied different concentrations of an agent to cells in triplicate experiments and monitored their effects with DIC-LM, OGA7-13^488^ labeling and both FESEM and TEM. We then chose those concentrations that caused lattice alterations but did not alter cell proliferation in culture (e.g., cell expansion and division). Second, most experiments were performed in media adjusted to pH 7.2, except for some enzyme treatments where the pH of the medium was adjusted to match the suppliers’ pH optimum specifications (e.g., pH 6.0, endoarabinanse, rhamnosidase, cellulose, xyloglucanase; pH 8.0, PME). As a control, we cultured cells without the enzymes at the different respective pHs and no differences in lattice formation were observed. Third, as a further control to account for the effects of any preservative/buffer agents in the commercial enzymes that might cause alterations, aliquots of enzymes were dialyzed against 3 liters of deionized water over 24 h before application. Likewise, aliquots of enzymes were placed in a boiling water bath for 5 min., cooled on ice, dialyzed as above and used in test experiments. The latter treatment caused no lattice alteration, indicating that any lattice changes were due to enzymatic activity. Finally, to verify that the experimental agents caused alterations, cells from all treatments were washed (see Materials and Methods) and allowed to recover. Recovery was then monitored using OGA7-13^488^ labeling

### General Architectural Design of the Pectin Lattice

**Figure 1:**
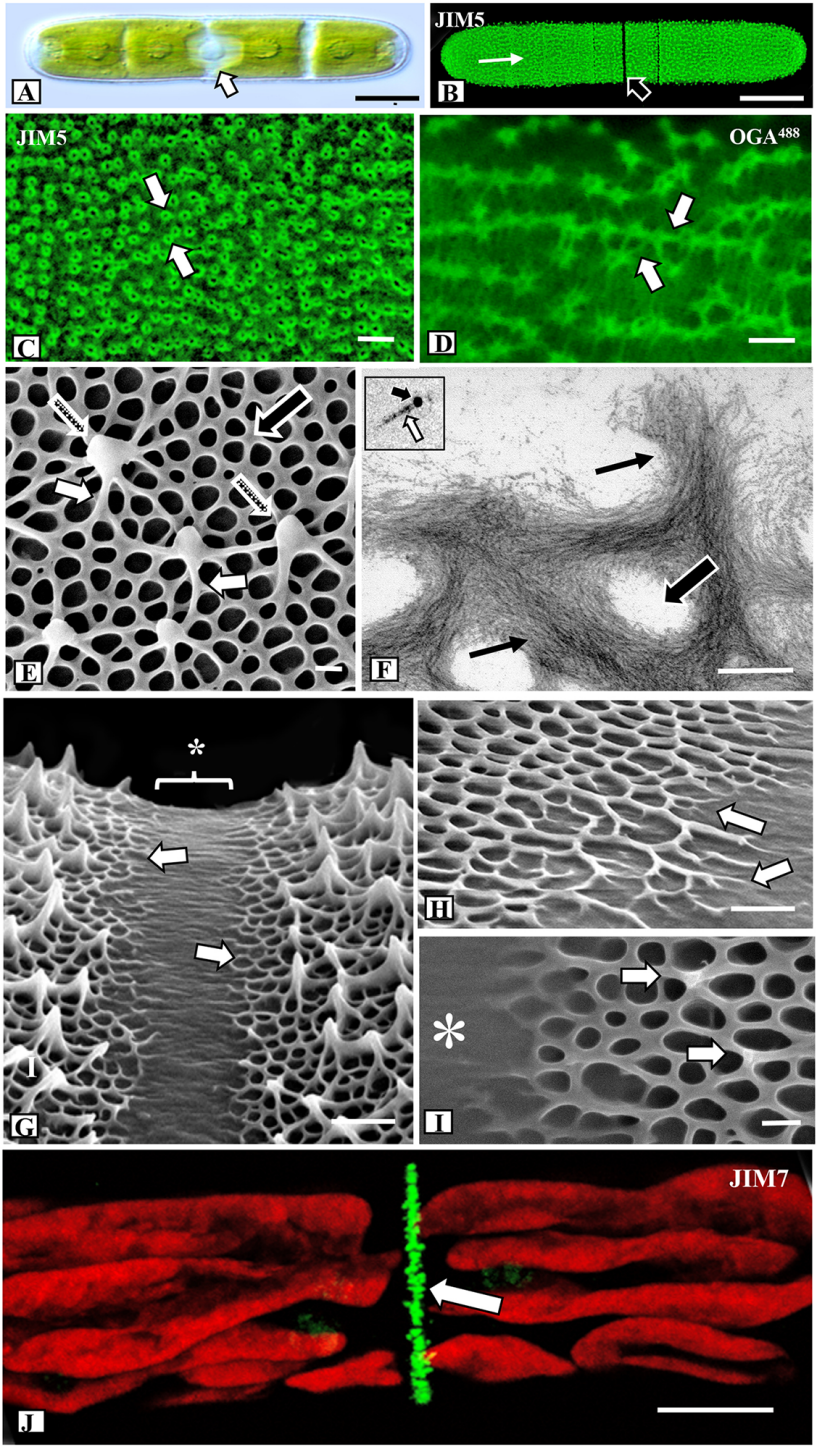
Microarchitecture of the pectin lattice. **(A)** Differential interference contrast (DIC) image of *Penium* showing its typical cylindrical shape and the central isthmus zone (arrow) where new cell wall material is deposited. Bar, 15 µm. **(B)** JIM5 (specificity: Homogalacturonan, HG, with low degree of methyl-esterification; Clausen et al., 2003) labeling of the HG lattice (white arrow) of a live cell. Note the lack of labeling at the isthmus (black arrow). Confocal laser scanning microscopy (CLSM) image. Bar, 15 µm. **(C)** Magnified view of the JIM5-labeled HG lattice. Note the fluorescently-labeled circular components (arrows) that represent the tips of the lattice projections. CLSM image. Bar, 22 nm. **(D) OGA7-13^488^** (specificity: and oligogalacturonide probe that binds to oligogalacturonide probe that HG with 7-13 contiguous GalAs; Mravec et al., 2017b) labeled HG lattice. Note the enhanced resolution of the fibers (arrows) that constitute the lattice. CLSM image. Bar, 25 nm. **(E)** Field emission scanning electron microscopy (FESEM) image of the HG lattice. Note the network of basal fibers (black arrow) that occasionally branch outward (small white arrows) to yield the thickened projections (stippled arrows). Bar, 215 nm. **(F)** Transmission electron microscopy (TEM) image of the HG lattice. Note the tight aggregation of thin fibrils (arrows) that form the basal fibers. Openings are present between the fibers (large arrow). Bar, 225 nm. Inset: HG fibril (white arrow) near 10 nm gold particle (black arrow). **(G)** FESEM image of the isthmus zone. The center of the isthmus (*) is smooth while at the two edges (arrows) the basal fibers emerge from the inner wall layers. Bar, 725 nm. **(H)** Magnified FESEM image of the branching basal fibers (arrows) emerging from the isthmus (arrows). Bar, 450 nm. **(I)** The formation of the lattice projections occurs approximately 250 nm from the edge of the isthmus (*). Bar, 225 nm. **(J)** JIM7 (specificity: Homogalacturonan, HG, with high degree of methyl- esterification; Clausen et al., 2003) labeling of high esterified HG deposition at the isthmus (arrow). CLSM image. Bar, 8 µm.

*Penium* is a unicellular placoderm desmid whose phenotype is an elongate cylinder with two rounded poles. Two to four chloroplasts fill each semicell (i.e., each of two equal cell halves) and surround the central isthmus zone that contains the nucleus (**Figure 1A**). The isthmus is the main site of cell wall and cell expansion (Domozych et al., 2014a). The cell wall surface is covered by the HG lattice, which was readily labeled with JIM5 (**Figure 1B**), a mAb that binds to relatively low esterified HG (Knox et al., 1990; Clausen et al., 2003). Closer examination revealed that JIM5 labeling was most intense near the tip of the outward-extending HG projections (**Figure 1C**), and the walls of each projection labeled while the central zone did not. OGA7-13^488^ (Mravec et al., 2017b), also labeled the HG lattice, revealing the network of basal fibers and their inclusive branches that emerged and fused to form the outer projections (**Figure 1D**). OG7-13^488^ is a fluorescently labeled oligogalacturonide that binds to HG with a degree of polymerization (DP) of 7-13. OGA7-13^488^ binds HG with a low degree of esterification, based on the complexation with calcium and the details of the specificity towards HG with different degree of esterification (Mravec et al., 2017b). This probe is much smaller and most likely penetrates and label HG-structures more easily than a mAb. Its size also allows for finer resolution of the pectin infrastructure and was therefore used for subsequent live cell labeling of the lattice.

For high resolution imaging of the HG architecture on the cell wall surface we used FESEM. Cells were collected from control, treated and recovery cultures and prepared for FESEM imaging using a freeze shattering technique (Wasteneys et al., 1997) and subsequent lyophilization (**Supplementary Figure 1A**). This technique allows for rapid preparation of cells for imaging of the lattice and caused little or no damage to the cell walls. These same walls could also be used for analysis with Energy Dispersive X-ray Spectroscopy (EDS). Furthermore, one of the valuable features of *Penium* for cell wall structural studies is that both the altered lattice zones caused by the specific treatments and the pre-treatment (unaltered or “regular”) lattice zones are usually found on the same cell. This allows for direct comparative analysis. For quantitative FESEM analyses of the HG lattice, we collected specific measurements from 50 different cells. The HG lattice was observed to consist of two major parts: (1) a network of basal fibers that covers the cell wall surface and (2) a series of outward facing projections resulting from the fusion of branches of the basal fibers that rise up over the wall surface (**Figure 1E**; **Supplementary Figure 1B**). In control cells, 70–75 (+/- 5) projections were found on every 100 µm^2^ transect of most the cell wall surface, with the exception of the isthmus where the HG lattice formed during expansion and the tips of the polar zones (**Supplementary Figure 1C**) where the projections were thicker and more closely spaced. The spacing between the peaks of the projections varied from 0.8 to 1.4 µm. The thickness of a basal fiber measured 60–70 nm (+/- 5 nm) and the fibers were observed to rise up between 150–200 nm (+/-10nm) above the wall surface. At junction zones, where the fibers merged to form the reticulated network, the thickness of the fiber increased to 125–150 nm (+/- 15 nm). The size of the holes varied considerably, with an edge-to-edge spacing of 200–275 nm (+/- 25 nm), and 4–6 branches of basal fibers were observed to periodically extend outward and fuse with adjacent branches to form the projections that rose up 500–700 nm from the surface. TEM imaging of the walls of cells treated with 25 mM EDTA for 10 min prior to fixation showed that the HG lattice fibers consisted of multiple thin parallel fibrils (**Figure 1F**, **Supplementary Figures 1D, E**), each measuring 5.0–6.0 nm in diameter (**Figure 1F** inset). In an analysis of 50 EDTA-extracted cell walls, we estimated that each basal fiber (outside a fused branch zone) comprises 250–300 fibrils. Fibrils from the inner part of the HG lattice penetrated the medial layer (**Supplementary Figures 1F–H**). The medial layer contained densely packed fibrils that formed narrow and branched tube-like incursions through the inner layer of cellulose (**Supplementary Figure 1F, G**). The fibrillar aggregates of the lattice, as well as the medial wall layer, labeled with JIM5 (**Supplementary Figure 1H**). In addition, the medial wall layer, but not the HG lattice, labeled with INRA-RU1, a mAb that is specific for the backbone of RGI [**Supplementary Figure1G**; (Ralet et al., 2010)].

Cell wall expansion occurs at the isthmus (Domozych et al., 2014a). FESEM imaging revealed that the isthmus zone was between 1.25 and 1.5 µm wide and was devoid of HG fibers (**Figure 1G**). At the edges of the isthmus, the HG fibers emerged from the underlying wall layer (**Figure 1H**). Typically, the first appearance of basal fibers was 500 nm (+/- 50 nm) from the center of the isthmus, while projections first appeared at 1.5–2.0 µm from the center of the isthmus (**Figure 1I**). The HG lattice and other cell wall components were first deposited at the isthmus. The new wall displaces older wall components toward the two poles. TEM imaging showed that the isthmus was devoid of the HG lattice (**Supplementary Figure 2A**). Immunogold labeling with INRA-RU1 showed initial labeling of the nascent medial layer (**Supplementary Figure 2B**) and JIM5 labeled the forming lattice near the isthmus edges (**Supplementary Figure 2C**). These observations suggest that the RG-1 component forms before the HG is deposited on the wall surface. Finally, labeling with JIM7 (specificity: Homogalacturonan, HG, with high degree of methyl- esterification; Clausen et al., 2003), showed a narrow band of punctate labeling at the isthmus (**Figure 1J**).

### Ca^2+^ Levels at Specific Lattice Loci

**Figure 2:**
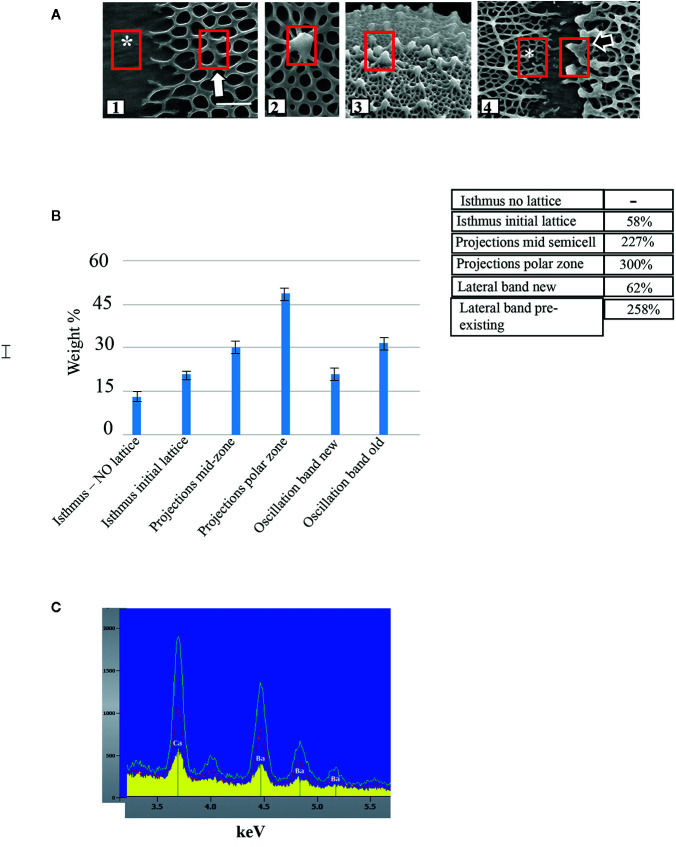
Comparison of levels of Ca^2+^-complexation at different parts of the lattice. **(A)** Geographic location of EDS measurement sites in untreated cells (red boxes) including: (1) isthmus (*) and peri-isthmus zone (arrow) where basal fibers arise; (2) lattice projection at the mid-semi cell; (3) lattice projection at a polar zone; (4) lateral band zone at both newly formed lattice (*) and pre-existing lattice (black arrow site). **(B)** Comparison of Ca^2+^ levels along specific loci of the cell wall. SE are shown. Rows show comparison of relative amounts of Ca^2+^ at specific cell wall loci. The lattice free zone in the mid-isthmus region was set as the baseline for comparison. For example, the projections at the mid -cell region yielded a signal 227% as strong as the lattice free zone. **(C)** Energy dispersive X-ray spectroscopy (EDS) profiles of wall surface of cells grown in WHM containing 1 mM CaCl_2_ and 5 mM BaCl_2_. Note the presence of both Ca^2+^ and Ba^2+^ in the wall and the increasing levels from the isthmus (yellow), mid-semicell (red) and polar zones (green).

We used EDS to compare the levels of Ca^2+^-complexation at different parts of the lattice. Six zones were monitored: the central isthmus zone where no lattice fibers were noted (**Figure 2A1**, *); the peri-isthmus zone where the basal meshwork of fibers first appear (**Figure 2A1**); the fully formed lattice in the zone between the isthmus and poles (i.e., mid-semicell **Figure 2A2**); the lattice of the polar zone (**Figure 2A3**); and the edges of the lateral bands that are found over the whole cell wall surface (**Figure 2A4**). For the latter, the edge containing earlier-forming lattice consists of thick projections, while the newly formed lattice edge consists of the basal fiber meshwork (*). For each zone, we examined three 0.5 x 0.5 µm transects in each of 10 cells and averaged the weight % of the signal. While the amounts of Ca^2+^ were not precisely quantified, we used the average measurements of each zone to compare the relative amounts of Ca^2+^ per zone (**Figure 2B**). After setting the lattice-free isthmus zone as a base for comparison, we found that the initial basal fiber meshwork contained approximately 58% more Ca^2+^ than the mid-isthmus zone. The typical lattice (i.e., basal fiber meshwork and projections) of mid-semi cells and lattice of the polar zone had 227% and 300% more calcium, respectively, than the mid-isthmus zone. The new basal fibers of the lateral bands had 62% more Ca^2+^ while the pre-existing zone of the band had 258% more Ca^2+^ than the mid-isthmus zone.

### Changes to Lattice Architecture in Response to Treatment With Chemical Agents

**Figure 4:**
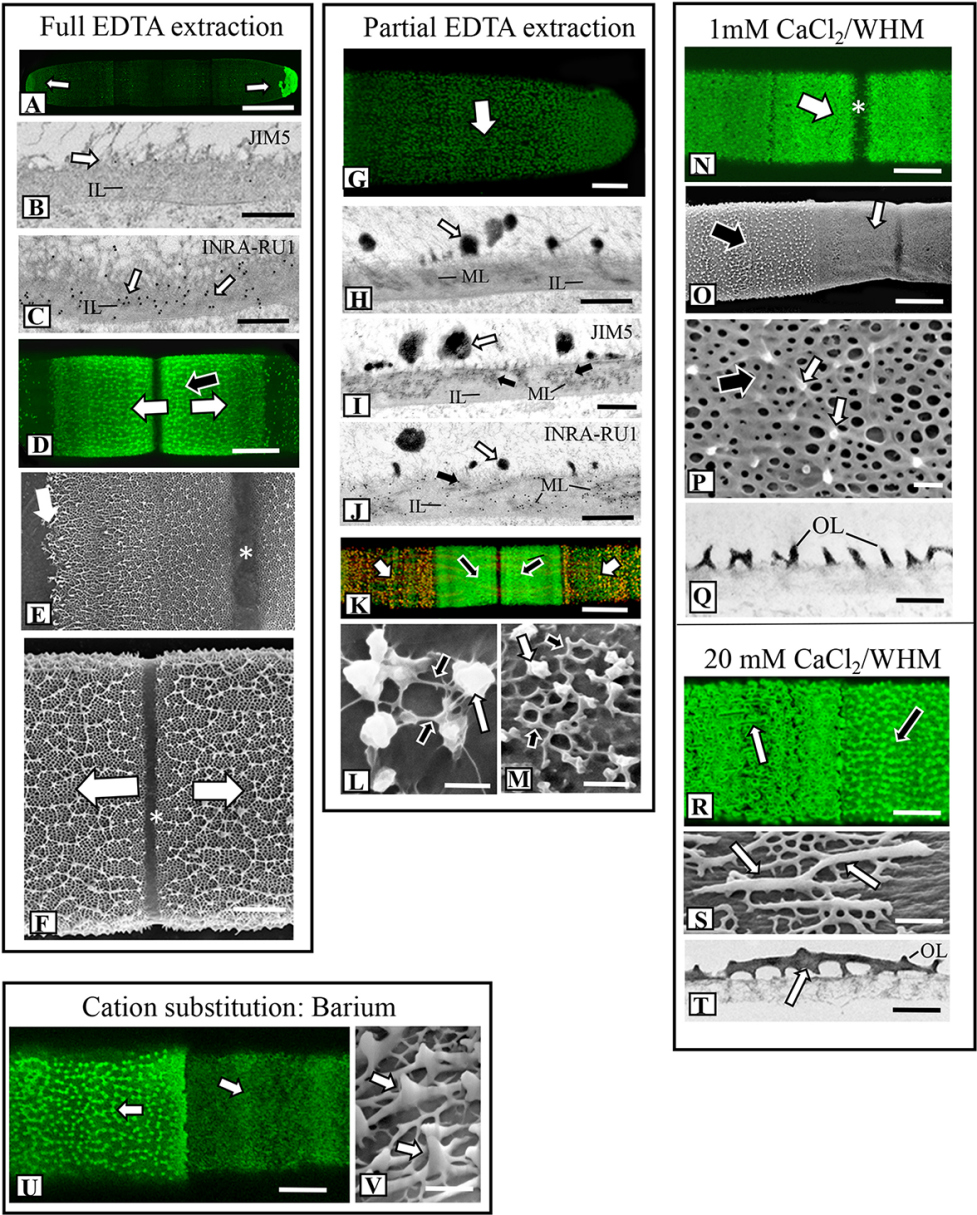
Fine structural details of lattice alterations in cells treated with chemical agents. **(A)** OGA7-13^488^ labeling of a cell incubated in 50 mM EDTA for 90 min (full extraction). The lattice is removed from the cell wall surface except for the polar zones (arrows). Confocal laset scanning microscopy (CLSM) image. Bar,15 µm. **(B)** JIM5-labeled TEM image of the cell wall of a cell from full EDTA extraction. The lattice has been removed (white arrow), leaving the inner layer (IL). The typical medial layer is not apparent and JIM5 labeling is virtually absent. Bar, 750 nm. **(C)** INRA-RU1 labeled TEM image of the cell wall of a cell from full extraction. Note the heavy labeling (white arrows) of the inner layer (IL). The lattice is absent as well. Bar, 750 nm. **(D)** OGA7-13^488^ labeling of the isthmus zone of a cell recovering for 48 h after full EDTA extraction. A new lattice forms at the isthmus zone (arrows) like that in untreated cells. CLSM image. Bar, 7.5 µm. **(E)** Field emission scanning electron microscopy (FESEM) image of a cell recovering for 48 h after full EDTA extraction. The initial lattice formed at the isthmus (white arrow) is less organized than the lattice that is subsequently formed (black arrow) at the isthmus zone (*). Bar, 1.6 µm. **(F)** Magnified FESEM image of the new lattice during later stages recovery after full EDTA extraction cell at the isthmus zone (*). Newly formed lattice displaces older lattice toward the two poles (arrows). Bar, 3 µm. **(G)** OGA7-13^488^ labeling of a cell that was treated with 50 mM EDTA for 10 min (i.e., partial extraction). Weakly fluorescent remnants of the lattice are visible (arrow). CLSM image. Bar, 6 µm. **(H)** TEM image of the cell wall of a cell after partial EDTA extraction. Remnants of the lattice remain (white arrow). The medial layer (ML) and inner layer (IL) are also apparent. Bar, 1 µm. **(I)** JIM5 labeled TEM image of the medial layer (black arrows) of the cell wall from partial EDTA treatment. Labeling is also noted on the lattice remnants (white arrow) Bar, 1 µm. **(J)** INRA-RU1 labeled TEM image of the medial layer (ML; black arrows) arrows) of the cell wall from partial EDTA treatment. The inner layer (IL) and lattice remnants are also noted. Bar, 800 nm. **(K)** Co-labeling of cell recovering from partial extraction for 48 h. JIM5-TRITC was assigned a red pseudo-color while OGA7-13^488^ was assigned a green pseudo-color. The overlap of the pseudo-colored probes represents wall remnants while green represents zones where new HG was deposited. Labeling is noted at both the isthmus (black arrows) and in scattered punctate found on the wall surface outside the isthmus (white arrows). CLSM image. Bar, 15 µm. **(L)** FESEM image of HG lattice reforming outside the isthmus 12 h after recovery from partial EDTA extraction. Note the thin fibers (black arrows) connecting the lattice remnants (white arrow). Bar, 450 nm. **(M)** FESEM image of HG lattice reforming outside the isthmus 24 h after recovery from partial EDTA extraction. Note the reformation of the basal fiber network (black arrows) in between the lattice remnants (white arrow). Bar, 450 nm. **(N)** OGA7-13^488^ labeling of the altered isthmus zone (white arrow, *) of a cell grown in low Ca^2+^-medium (1 mM CaCl_2_ in WHM) for 4 days. Bar, 8 µm. **(O)** FESEM image showing the distinct changes at the isthmus (white arrow) in a cell grown in low Ca^2+^-medium. Bar, 8 µm. **(P)** Magnified FESEM image of the lattice formed under low Ca^2+^ conditions. The underlying basal fiber meshwork remains (black arrow) but only a few projections form (white arrows). Bar, 400 nm. **(Q)** TEM image of the lattice of a cell grown under low Ca^2+^ conditions for 4 days. Note the highly reduced projections on the outer layer (OL). Bar, 750 nm. **(R)** OGA7-13^488^ labeling of a cell grown under high Ca^2+^ conditions (20 mM Ca^2+^ in WHM). Note the elongate fibers at the isthmus zone (arrow). CLSM image. Bar, 4 µm. **(S)** FESEM image showing the altered lattice in a cell grown under high Ca^2+^ conditions. Thick, branched and elongate projections (arrow) emerge from the few basal fibers on the outer wall layer. Bar, 800 nm. **(T)** TEM image of the thickened projections (arrow) on the outer layer (OL) grown under high Ca^2+^ conditions. Bar, 850 nm. **(U)** OGA7-13^488^ labeling of the altered lattice at the isthmus zone (white arrow) of a cell grown with 5 mM barium BaCl_2_ in WHM with 1 mM CaCl. CLSM image. Bar, 16 µm. **(V)** FESEM image of the altered lattice showing the thickened fibers (arrows) in a cell grown in BaCl_2-_containing medium. Bar, 220 nm.

**Figure 3:**
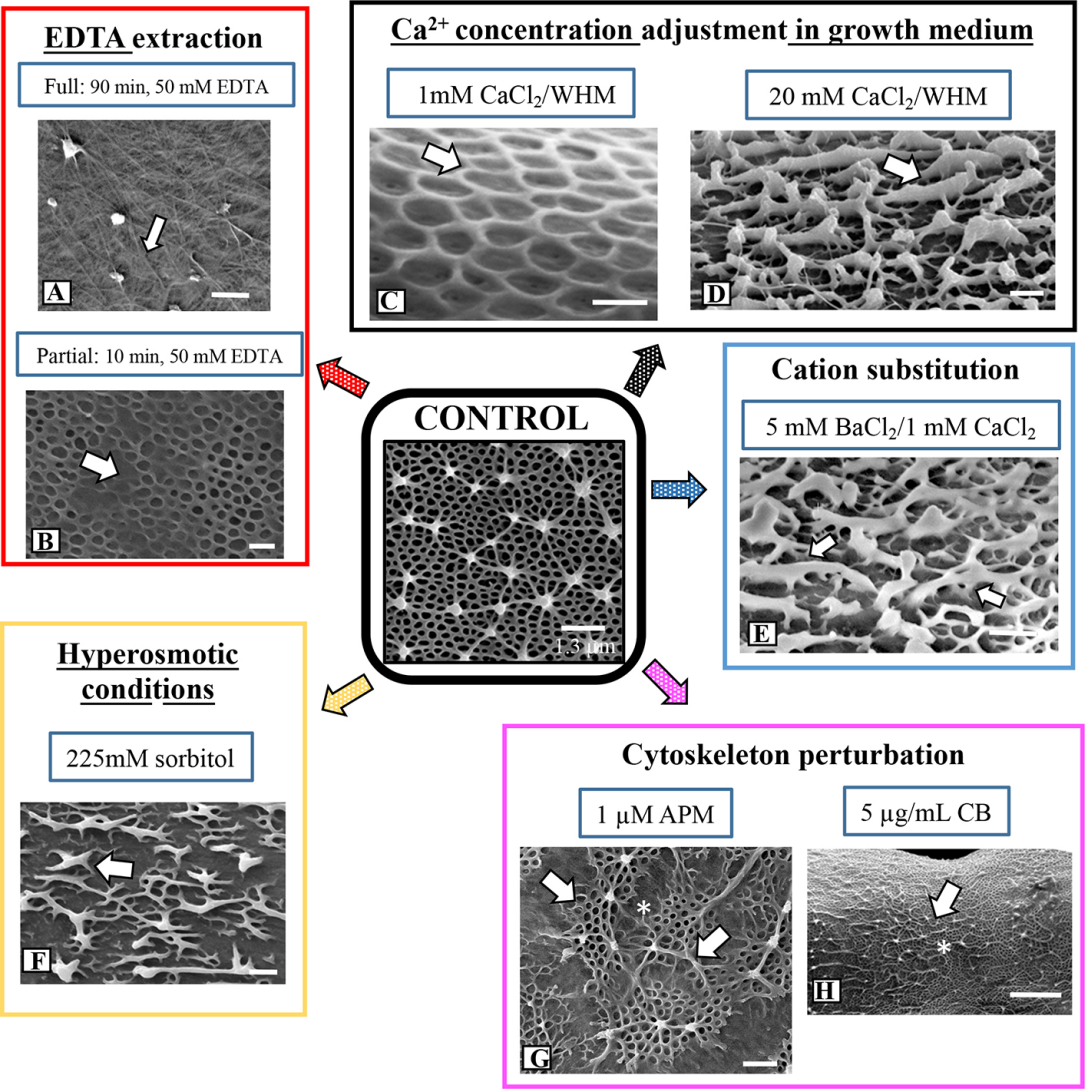
Summary of architectural changes to lattice upon chemical and physical stress. (**A)** Treatment with 50 mM EDTA for 90 min removes the lattice leaving the inner wall layer containing cellulose microfibrils (arrow). Bar, 300 nm. **(B)** Partial EDTA extraction (50 mM EDTA for 10 min) removes most of the lattice projections but leaves remnants of the basal fiber network (arrow). Bar, 420 nm. **(C)** When cells are grown for 5 days in low Ca^2+^-medium (1 mM CaCl_2_ in WHM), the lattice projections do not form. Only the basal fiber network remains (arrow). Bar, 215 nm. **(D)** When cells are grown in high Ca^2+^-medium (20 mM CaCl_2_ in WHM) for 5 days, highly thickened, ridges emerge (arrow) off the basal fiber network. Bar, 210 nm. **(E)** When cells are grown in low Ca^2+^-medium (1 mM CaCl_2_ in WHM) supplemented with 5 mM BaCl_2_, the lattice is transformed to irregularly-spaced and thickened fibers (white arrows) with little of the basal fiber network. Bar 250 nm. **(F)** Disruption of the lattice (arrow) in a cell grown in hypertonic conditions (225 mM sorbitol) for 4 days. Bar, 250 nm. **(G)** The irregular patch-like lattice (arrows) at the swollen isthmus zone (*) in a cell grown in 1 µM APM (amiprophos-methyl) for 48h. Bar, 500 nm. **(H)** The lattice of a cell grown in 8 µg/ml of CB for 4 days exhibits few alterations (arrow) at the isthmus (*) when compared to control cells. Bar, 1.3 µm.

We used FESEM as the primary tool for investigating topographic changes to the pectin lattice when cells were exposed to a variety of stress agents. Our focus was at and around the isthmus, i.e., the site of new wall expansion. **Figure 3** shows a summary of the results of lattice changes in response to interrogation with an array of chemical agents. When incubated in a 50 mM solution of the calcium chelator EDTA for 90 min, the lattice was nearly totally absent (**Figures 3A** and **4A**; i.e., full extraction), exposing the underlying network of cellulose microfibrils of the inner wall layers. The cylindrical shape of the cell did not change when the lattice is removed. TEM-immunogold analysis of the fully extracted wall showed that the lattice was removed, leaving only the inner layers (**Figure 4B**). INRA-RU1 (specificity: Rha-(1,4)-GalA-(1,2)-Rha-(1,4)-GalA-(1,2)-Rha-(1,4)-Rha-(1,4)-GalA-(1,2)-Rha-(1,4)-GalA-(1,2)-Rha-(1; Ralet et al., 2010) labeling of the inner layers demonstrated that the RGI component remained (**Figure 4C**), but that the fine structure of the medial layer was no longer present. After 48 h of recovery, the lattice reformed at the isthmus and was indistinguishable from that of untreated cells (**Figure 4D**). FESEM imaging revealed that the lattice that was formed immediately (0–2 h) at the start of recovery was notably disorganized (**Figure 4E**), while longer recovery periods (24 h) yielded a typical lattice, as seen in control cells (**Figure 4F**).

When cells were incubated in 50 mM EDTA for 10 min (i.e., partial extraction), the HG lattice was devoid of projections and present as much-reduced basal fiber network (**Figures 3B** and **4G**). TEM imaging revealed that the inner and medial layers remained (**Figure 4H**), as did a few projections. JIM5 labeled the medial layer and remnant projections (**Figure 4I**) and INRA- RU1 the medial wall (**Figure 4J**). When cells were allowed to recover, lattice labeling with OGA7-13^488^ was apparent, both at the isthmus and at the other regions of the cell wall surface (**Figure 4K**). Here, we first labeled treated cells with JIM5-TRITC (a red pseudo-color assigned for fluorescent signal), allowed them to recover for 48 h and then labeled with OGA7-13^488^ (a green pseudo-color assigned for fluorescent signal). HG lattice components that were remnants of ‘old’ HG appeared in orange (i.e., overlap of the red initial JIM5-TRITC label and the green OGA7-13^488^ label). New HG components, labeled in green, were found at the isthmus and zones external to the lattice. FESEM imaging of the non-isthmus zone showed that after 12h recovery, narrow fibers connected the lattice remnants (**Figure 4L**). After 24 h, the typical basal meshwork appeared around the lattice remnants (**Figure 4M**).

### Changes in the Ca^2+^ Level in Growth Medium or Addition of Ba^2+^ Alter Lattice Formation

We observed that the formation of the pectin lattice is sensitive to levels of Ca^2+^ in the growth medium (i.e., the concentration of CaCl_2_). When cells were grown for 4 days in low Ca^2+^ concentrations (1 mM vs. 3 mM in regular WHM), the basal fiber network was still present, but the projections were no longer observable (**Figure 3C**). OGA7-13^488^ labeling of live cells and subsequent FESEM imaging of these treated cells highlighted significant alterations in the lattice at the isthmus (**Figures 4N, O**). In cells incubated for shorter periods of time (**Figure 4P**), very short projections emerged from some parts of the basal fiber network. TEM imaging confirmed that the lattice was much reduced (**Figure 4Q**). Cells incubated in WH medium with 20 mM of Ca^2+^ (high Ca^2+^ medium) produced a lattice that consisted of thickened, elongate projections (**Figures 3D** and **4R, S**), instead of the typical branched basal fiber network. TEM imaging highlighted the thickening of the projections (**Figure 4T**).

To determine the effects of the application of another divalent cation, Ba^2+^, in growth medium, we grew cells in WHM containing 1 mM CaCl_2_ and 5 mM BaCl_2_. We chose these conditions because cells grew poorly in medium without CaCl_2_ and with BaCl_2_. Addition of a small amount of CaCl_2._ (1mM) yielded typical cell numbers and also growing in high Ba^2+^ conditions. Under these conditions, FESEM imaging and OGA7-13488 labeling showed distinct alterations of the lattice (**Figures 3E** and **4U**). This was manifested as a few, irregularly spaced basal fibers that occasionally gave rise to highly thickened projections (**Figure 4V**). EDS examination showed that Ba^2+^ was taken up by the lattice (**Figure 2C**).

### Hyperosmotic Conditions Affect Deposition of HG Lattice

**Figure 5:**
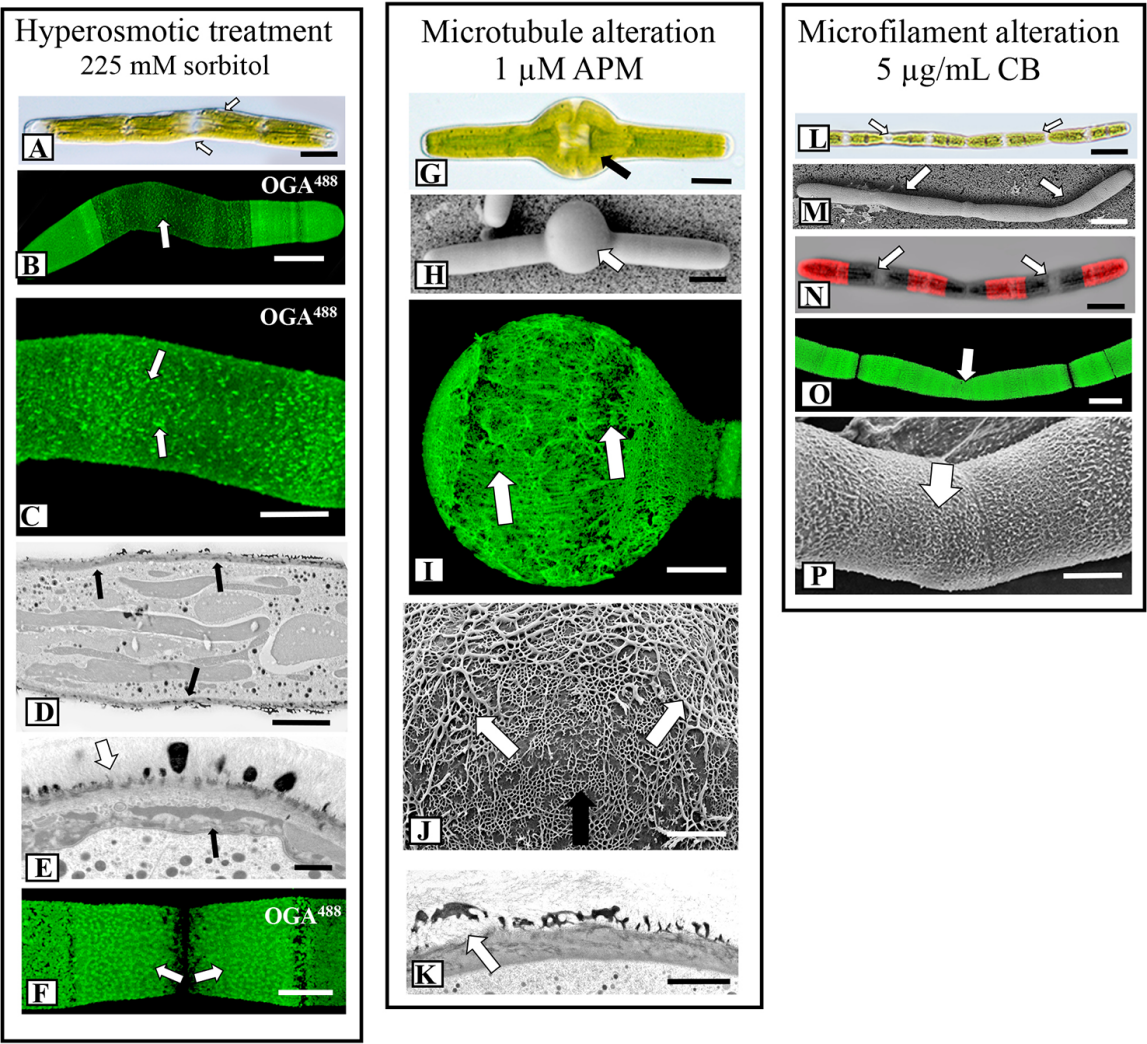
Fine structural details of lattice alterations. **(A)** Cell shape changes (arrows) during incubation hypertonic medium (225 mM sorbitol in WHM; 4 days). DIC image. Bar, 15 µm. **(B)** OGA7-13^488^ labeling of cell grown in hypertonic medium. The cell shape significantly changes and the lattice is notably reduced at the isthmus zone (arrows). Confocal laser scanning microscopy (CLSM) image. Bar, 15 µm. **(C)** Magnified view of an OGA7-13^488^ labeled cell grown in hypertonic medium showing that the lattice at the isthmus is reduced to irregular-spaced components on the cell wall surface (arrows). Bar, 8 µm. **(D)** Transmission electron microscopy (TEM) image of the irregular cell wall (arrows) formed during incubation in hypertonic medium. The lattice appears in irregular patches. Bar, 6 µm. **(E)** Magnified TEM image of the altered cell wall of a cell grown in hypertonic medium. The lattice components are irregular (white arrow) and just inside the plasma membrane are unique, multi-component components (black arrow). Bar, 1 µm. **(F)** Upon recovery (48 h) from incubation in hypertonic growth medium, a new lattice forms at the isthmus (arrows), similar to that observed in control cells.OGA7-13^488^ labeled cell. CLSM image. Bar, 8 µm. **(G, H)** When cells are incubated in 1 µM APM (amiprophos-methyl) for 36 h, notable swelling occurs at the isthmus (arrow). **(G)** = DIC image; **(H)**, Field emission scanning electron microscopy (FESEM) image. **(G)**, Bar, 17 µm. **(H)**, Bar, 15 µm. **(I)** OGA7-13^488^ labeling of a cell incubated in APM for 48 h. Note the disruption of the HG lattice in the swollen isthmus zone (white arrow) and the formation of elongate thickened fibers. CLSM image. Bar, 15 µm. **(J)** FESEM image of the interface of the pre-existing HG lattice (black arrow) and the altered lattice (white arrow) of the swollen isthmus zone formed during APM incubation. Note the disorganized thick elongated fibers of this altered zone. The pre-treatment wall is also observed (black arrow). Bar, 800 nm. **(K)** TEM image of the altered lattice (arrow) of an APM treated cell. Bar, 1µm. **(L, M)** The formation of elongate “filamentous” phenotypes when cells are treated with 8 µg of CB (cytochalasin B) for 48 h. Individual cell units are apparent (white arrows). **(L)**, DIC image, **(M)** FESEM image. **(L)**, Bar, 30 µm; **(M)**, Bar, 30 µm. **(N)** CB- treated cell that was first labeled withOGA7-13^488^ and then placed back in culture for 24 h. Note that wall expansion and HG lattice formation still occur (arrows). CLSM image. Bar, 30 µm. **(O)** OGA7-13^488^ labeled cell that was treated with CB. Note that the typical HG lattice remains almost intact (arrows). CLSM image. Bar, 17 µm. **(P)** FESEM image of the HG lattice of a cell grown with CB. Few changes to the lattice (arrow) are noted. Bar, 8.5 µm.

The lattice architecture also significantly changed when the cells were grown for 4 days in hyperosmotic medium (225 mM sorbitol in WHM; **Figure 3F**) and FESEM imaging revealed that it was comprised of irregularly spaced aggregates of the basal fibers. Branches from these fibers occasionally rose up, fused and formed thick elongate extensions rather than the typical punctate projections. We also noted distinct differences in cell shape under hyperosmotic conditions (**Figures 5A, B**): cells continued to expand, but bend at the region around the isthmus zone. Here, OGA7-13^488^ labeling revealed that the HG lattice was significantly disrupted (**Figure 5B**) and contained irregularly spaced fluorescent “areas” (**Figure 5C**). TEM analysis of the altered zone showed changes to both general wall structure and the lattice (**Figures 5D, E**). The wall at the altered zone contained irregular lattice components (**Figure 5E**), as well as distinct inclusions between the inner surface of the wall and the plasma membrane. During recovery, a new HG lattice formed at the isthmus (**Figure 5F**) in a manner similar to untreated cells.

### Effects of Microtubule- and Microfilament-Disrupting Agents on HG Lattice Formation

To investigate the role of the cytoskeleton in lattice formation, we treated cells with the microtubule disrupting agent, APM (amiprophos-methyl), 1.0 µM), and the actin- disrupting agent, cytochalasin B (CB; 5 µg/ml) for 12 h–4 days. APM treatment caused major swelling of the cell and significant changes to the lattice at the isthmus zone (**Figures 3G** and **5G, H**). OGA7-13^488^ labeling of live cells revealed major disruptions of the lattice (**Figure 5I**) and FESEM imaging showed that the swollen zone contained an irregular aggregation of the typical basal fiber network and unusual thick fibers on the wall surface (**Figure 5J**). TEM imaging of the swollen zone also revealed the irregular HG lattice and notable disorganization and size reduction of the medial layer (**Figure 5K**). APM disrupts microtubules in plants, (Falconer and Seagull, 1987) which in turn affects the deposition of cellulose microfibrils in the cell wall. We used the cellulose-label, CBM64a-GFP to monitor the effects of APM and saw that the cellulose-rich inner layers were significantly altered (**Supplementary Figures 3A, B**). In the swollen region, fluorescence was diminished significantly.

When cells were treated with CB for 4 days, only minor changes to the lattice were apparent (**Figure 3H**). However, during this treatment the cells expanded to form filaments (**Figures 5L, M**); that is, cell expansion continued but cytokinesis was inhibited or significantly altered, leading to an elongated cell made up of cell “units”, each with its own nucleus and isthmus zone. This was evident when these cells were labeled with OGA7-13^488^ (**Figure 5N**) and imaged with both fluorescence and DIC (Differential Interference Contrast microscopy). OGA7-13^488^ labeling of live cells revealed little alteration of the HG lattice (**Figure 5O**). Examination of the cell wall surface with FESEM showed that the HG lattice remained mainly intact (**Figure 5P**), although the outer projections displayed more irregular curling. During recovery from the APM and CB treatments, new lattice formation occurred at the isthmus and expands outward (**Supplementary Figures 4A, B**).

### Effects of Pectin-Targeted Enzymes on Lattice Architecture

**Figure 6:**
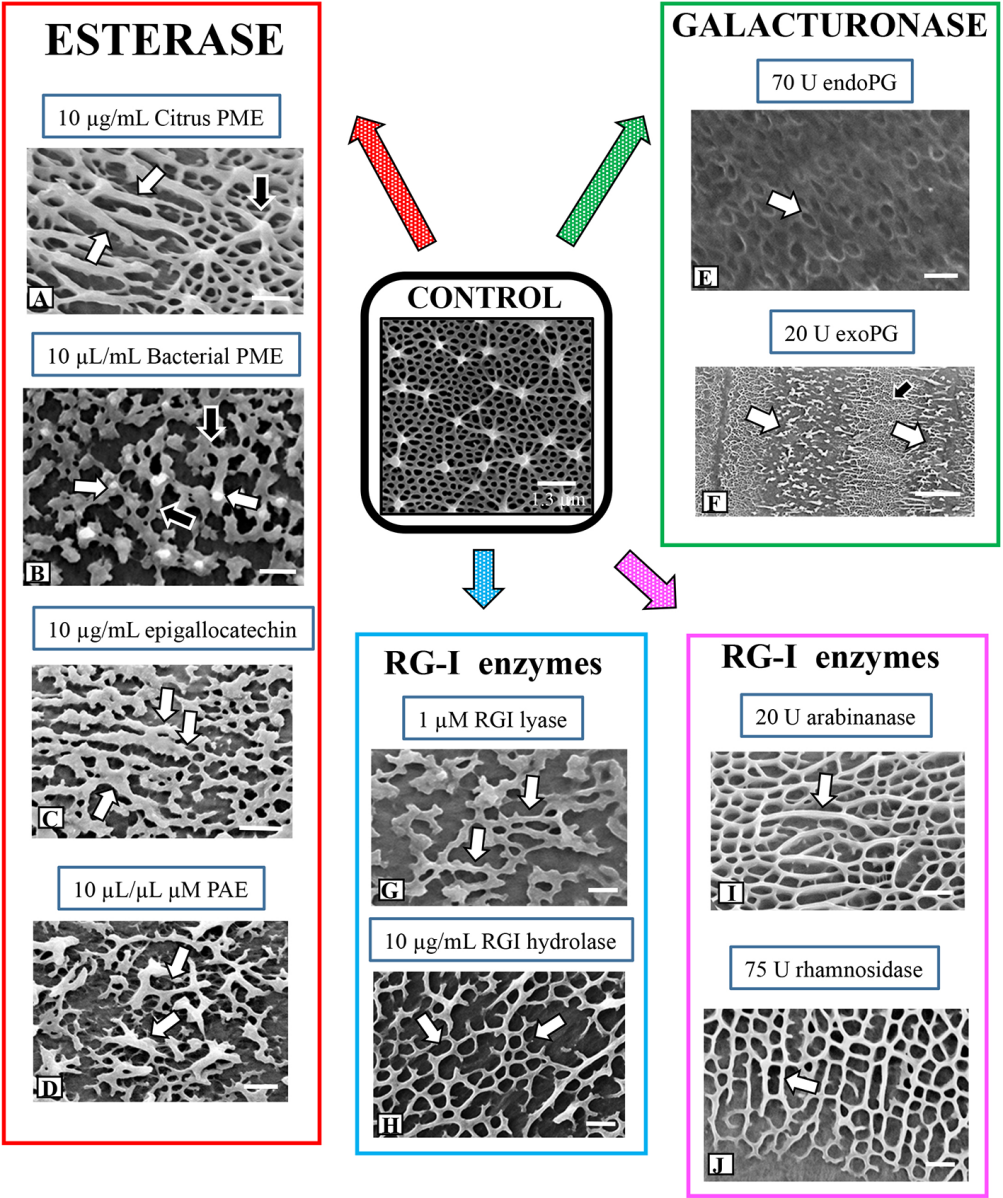
Summary of alterations to lattice microarchitecture in response to interrogation with pectin-targeting enzymes. **(A)** Treatment with citrus pectin methylesterase (PME) for 3 days results in the formation of elongate thickened ridges (white arrows) instead of the point-like projections (black arrow) found in pre-treatment walls or walls of control cells. Bar, 450 nm. **(B)** Treatment with bacterial PME for 3 days produces a network of flattened basal elements (black arrows) that occasionally yield small projections (white arrows). Bar, 250 nm. **(C)** Treatment with epigallocatechin for 4 days produces highly flattened basal elements (white arrows). Bar, 450 nm. **(D)** Treatment with bacterial PAE for 3 days yields an irregular network of flattened fibers with thickened projections (white arrows). Bar, 350 nm. **(E)** Cells grown in endoPG for 48 h produce cell walls with a much reduced basal fiber network (arrow) and no projections. Bar, 500 nm. **(F)** Cells grown in exoPG for 48 h produce cell walls with a bands of the typical lattice (black arrow) interspersed with zones consisting of irregular thickened elements (white arrows). Bar, 2 µm. **(G)** In cells grown in rhamnogalacturonan I (RGI) lyase for 4 days, the basal fiber network is present (arrow) but appears in patches with occasional thickened projections. Bar, 300 nm. **(H)** Field emission scanning electron microscopy (FESEM) imaging of a cell incubated with RGI hydrolase shows similar alterations (arrows) to the lattice as those observed with RGI lyase. Bar, 300 nm. **(I)** When cells are treated with arabinanase for 4 days, elongate and thickened ridges (arrow) project above the basal fiber network but no punctate projections are noted. Bar, 300 nm. **(J)** In cells treated with rhamnosidase, the basal fibers network remains but no projections are observed (arrow). Bar, 300 nm.

**Figure 7:**
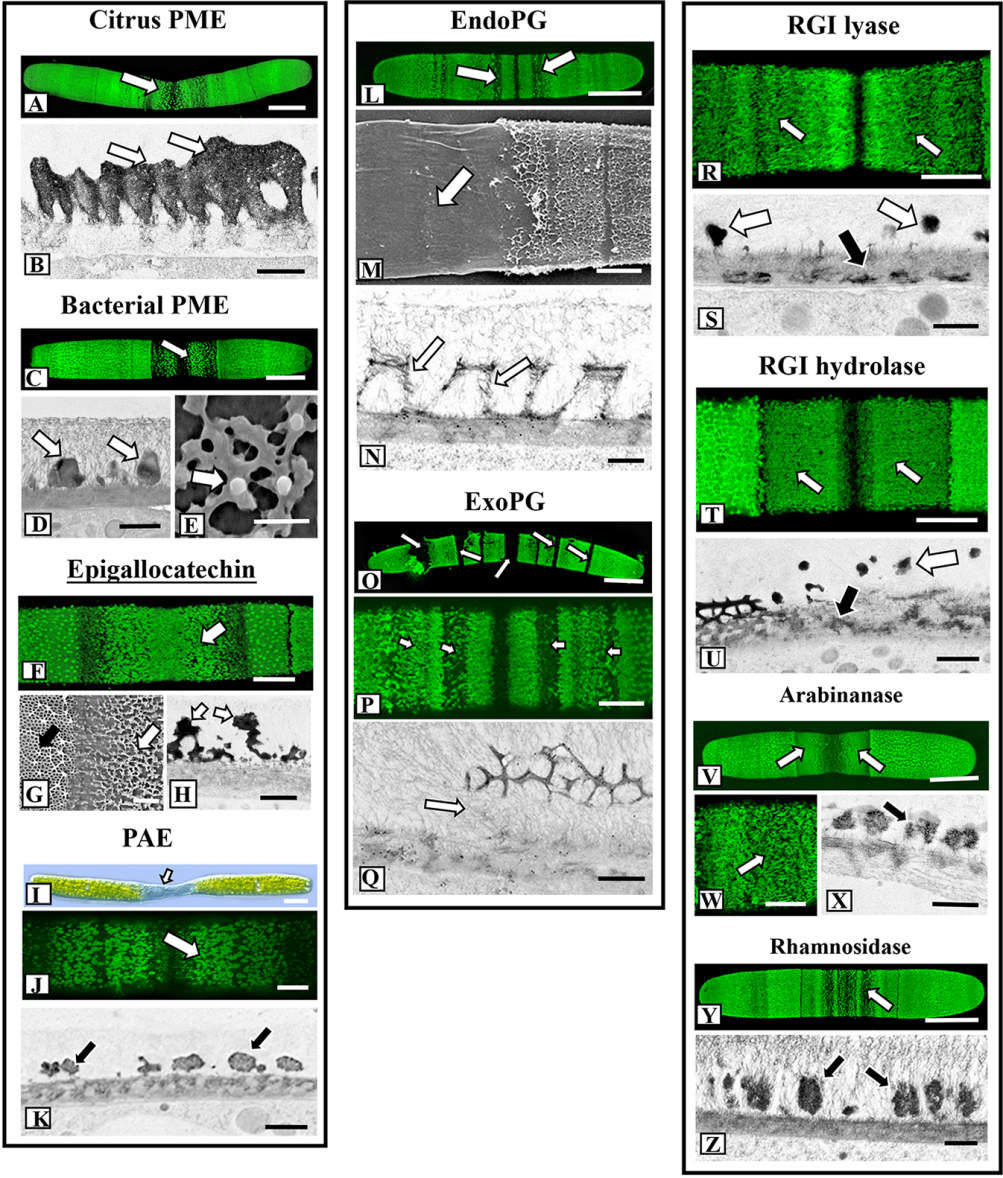
Fine structural details of lattice alterations during enzyme treatment. **(A)** OGA7-13^488^ labeling of the lattice alterations at the isthmus (arrow) of a cell treated with citrus pectin methylesterase (PME) for 4 days. Confocal laser scanning microscopy (CLSM) image. Bar, 17 µm. **(B)** Transmission electron miscroscopy (TEM) image of the highly thickened lattice (arrows) of the cell wall of a cell incubated in citrus PME. Bar, 500 nm. **(C)** OGA7-13^488^ labeling of the altered lattice (arrow) at the isthmus of a cell treated with bacterial PME for 4 days. CLSM image. Bar, 15 µm. **(D)** TEM image of the altered lattice of bacterial PME-treated cells reveals thick irregular lattice components (arrows). Bar, 400 nm. **(E)** Magnified field emission scanning electron microscopy (FESEM) image of the altered lattice formed during treatment with bacterial PME. Note the irregular flattened basal fibers with some producing a small emergent projection (arrow). Bar, 300 nm. **(F)** OGA7-13^488^ labeling of the altered lattice (arrow) at the isthmus of a cell treated with epigallocatechin CLSM image. Bar, 9 µm. **(G)** FESEM image of the distinct interface of the altered lattice (white arrow) vs. the regular lattice (black arrow) of the pre-treatment wall. Bar, 1 µm. **(H)** TEM image of the altered lattice (arrow) of an epigallocatechin-treated cell. Note the irregular thickened lattice components. Bar, 800 nm. **(I)** Differential interference contrast (DIC) image of a cell treated for 4 days in pectin acetylesterase (PAE). Note the expansion of the isthmus zone (arrow). Bar, 17 µm. **(J).** OGA7-13^488^ labeling of the altered lattice (arrow) at the isthmus of a cell treated with bacterial PAE. CLSM image. Bar,8 µm. **(K)** TEM image of the irregular and scattered lattice components (arrow) of a cell treated with bacterial PAE. Bar, 900 nm. **(L)** OGA7-13^488^ labeling of the altered lattice (arrows) of a cell treated with endoPG for 24 h. Note that the altered lattice appears in bands. CLSM image. Bar, 17 µm. **(M)** FESEM image of a cell treated with endoPG for 3 days. Note that the isthmus zone is devoid of a lattice (arrow). Bar, 4 µm. **(N)** TEM image of a cell treated with endoPG for 24 h. The lattice fibers are much thinner and consist of thin aggregates of fibrils (arrows). Bar, 750 nm. **(O)** TEM image of a cell treated with exoPG for 24 h. The lattice “peels” away from the wall surface (arrows). Bar, 17 µm. **(P)** Magnified image ofOGA7-13^488^ labeling of cell treated with exoPG for for 4 days. The altered lattice is in bands (arrows) that alternate with bands of unaltered wall. Bar, 8 µm. **(Q)** TEM image of a cell incubated in endoPG for 1 day. Note that the lattice appears to “peel off” the inner wall layers (arrow). Bar, 750 nm. **(R)** OGA7-13^488^ labeling of the altered lattice (arrows) of a cell treated with RGI lyase for 5 days. CLSM image. Bar, 8 µm. **(S)** TEM image of the cell wall of the isthmus of a RGI lyase treated cell wall. Note that the medial layer is much reduced (black arrow) at the wall surface and irregular distribution of lattice projections (white arrows). Bar, 750 nm. **(T)** OGA7-13^488^ labeling of the altered lattice (arrows) of a cell treated with RGI hydrolase for 5 days. CLSM image. Bar, 8 µm. **(U)** TEM image of the cell wall of the isthmus of a RGI hydrolase-treated cell wall. Note that the medial layer (black arrow) is much reduced at the wall surface and irregular distribution of lattice projections (white arrow). Bar, 750 nm. **(V)** OGA7-13^488^ labeling of the altered lattice (arrows) of a cell treated with arabinanase for 5 days. CLSM image. Bar, 17 µm. **(W)** Magnified view of the reduced isthmus lattice (arrow) in an arabinanase treated cell.OGA7-13^488^ labeling. Bar, 8 µm. **(X)** TEM image showing the alteration of the lattice (arrow) in a cell treated with arabinanase. Bar,750 nm. **(Y)** OGA7-13^488^ labeling of the altered lattice (arrow) of a cell treated with rhamnosidase for 5 days. CLSM image. Bar, 17 µm. **(Z)** TEM image of the altered lattice (arrows) in a rhamnosidase-treated cell. Bar, 1 µm.

A large and diverse group of pectin-targeting enzymes is critical to the biosynthesis and modulation of this cell wall polysaccharide (Sénéchal et al., 2014). In this study, we cultured cells in medium containing plant-derived PME (i.e., orange PME; de-esterifies in block-like fashion), bacteria-derived PME (from *Dickeya dadantii*; de-esterifies in random or non-block fashion*)*, pectin acetylesterase (PAE), and the PME inhibitor, epigallocatechin (Lewis et al., 2008), and monitored their effects on the HG lattice. Plant-derived PME treatment resulted in a much reduced basal fiber network with thick ridge-like “projections (**Figure 6A**; see also Domozych et al., 2014a). OGA7-13^488^ labeling of live cells confirmed the alteration to the lattice at the isthmus zone (**Figure 7A**). TEM imaging highlighted the thick elongate projections of the altered lattice (**Figure 7B**). The effects of incubation with bacterial PME on the lattice were notably different, as we saw patches of flattened basal fibers that occasionally yielded small projections (**Figure 6B**). These alterations were observable at the isthmus of live OGA7-13^488^-labeled cells as well as in TEM profiles (**Figures 7C, D**). Magnified FESEM imaging revealed that the basal fibers became broadly flattened, irregularly branched (**Figure 7E**) and occasionally yielded projections. Care must be taken in interpreting the results for these PMEs and other enzymes as alterations could be due to impurities in the enzymes stock solutions. To address this we dialyzed enzyme solutions to remove impurities before application and tested boiled (i.e., inactive) enzymes to ascertain if non-enzymatic components of the enzyme solutions caused any alterations (see *General Experimental Considerations* in *Results*). We also tested the effects of epigallocatechin, a blocker of PME activity (Lewis et al., 2008) on lattice formation. When incubated in medium containing epigallocatechin, no projections were observed, and the basal fibers were irregularly branched and flattened (**Figure 6C**). These distinct changes to the lattice were also observed following OGA7-13^488^ labeling and TEM/FESEM imaging (**Figures 7F–H**).

Some pectins have been shown to contain acetyl esters that are removed with pectin acetylesterase or PAE (Orfila et al., 2012; Sénéchal et al., 2014). At present, acetylation of *Penium*’s HG is unknown but we applied PAE to see if changes occurred to the lattice. When cells were incubated with exogenous PAE, the lattice consisted of irregular and small patches of thickened basal fibers (**Figure 6D**). During PAE treatment, the isthmus zone narrowed considerably, resulting a distinct, highly elongated phenotype (**Figure 7I**). OGA7-13^488^ labeling and TEM imaging (**Figures 7J, K**) further elucidated the changes to the lattice at the isthmus zone

### Lattice Alteration Upon Incubation with Endo (endoPG)- and Exopolygalacturonase (exoPG)

When cells were incubated in medium containing either endoPG or exoPG for 4 days, the HG lattice was significantly altered. endoPG treatment resulted in the complete loss of the lattice projections, leaving a significantly reduced basal fiber network (**Figure 6E**). During brief treatments of 24–48 h, these changes were seen at the isthmus, but also at bands throughout the cell surface (**Figure 7L**). During longer incubation periods (3–4 days), the HG lattice was completely removed from the wall surface (**Figure 7M**). TEM imaging of a cell treated for 3 days showed that the lattice appeared to disassemble into small aggregates of fibers (**Figure 7N**). exoPG treatment removed large stretches of the lattice, most notably in a series of bands (**Figures 6F** and **7O, P**). These bands contained irregular patches of thickened fibers that alternated with bands of the typical lattice. TEM imaging of the affected areas suggested that the lattice peeled away from the cell wall surface at these zone (**Figure 7Q**).

### Enzymes That Target RGI Have Minor Effects on Lattice Development

In our previous TEM analyses of the *Penium* cell wall, we noted that a medial wall layer, which consists of branched zones of packed electron dense fibers, was connected both to the HG lattice and the inner layer containing cellulose microfibrils (Domozych et al., 2014a). This medial layer labeled with the RGI-binding mAb, INRA-RU1 (**Supplementary Figure 3C**). In this current study, we incubated cells in medium containing enzymes that target RGI. Live cell labeling with OGA7-13^488^ after treatment with RGI lyase and RGI hydrolase (**Supplementary Figures 3C–E**) demonstrated disruption of the medial wall layer (**Supplementary Figures 3D, E**). High resolution imaging of alterations to the lattice revealed considerably fewer changes in architecture than were observed after treatment with HG modifying enzymes. When treated with RGI lyase or RGI hydrolase for 4 days, the basal fiber network was less branched (**Figures 6G, H**), more thickened and contained no projections. OGA7-13^488^ labeling showed that the alterations predominated at the isthmus (**Figures 7R, T**). TEM imaging revealed that the lattice was notably disrupted, as were the outermost regions of the medial layer (**Figures 7S, U**). Incubation of cells in arabinanase and rhamnosidase, enzymes that affect the side chains of RGI, also resulted in minor changes in the lattice (**Figures 6I, J**), where outer projections were not formed but a thickened basal fiber network was present. These results were confirmed by both OGA7-13^488^ labeling and TEM imaging of live cells (**Figures 7V, W, Y**) and TEM (**Figures 7X, Z**).

### Cellulase Treatment Also Disrupts the HG Lattice

**Figure 8:**
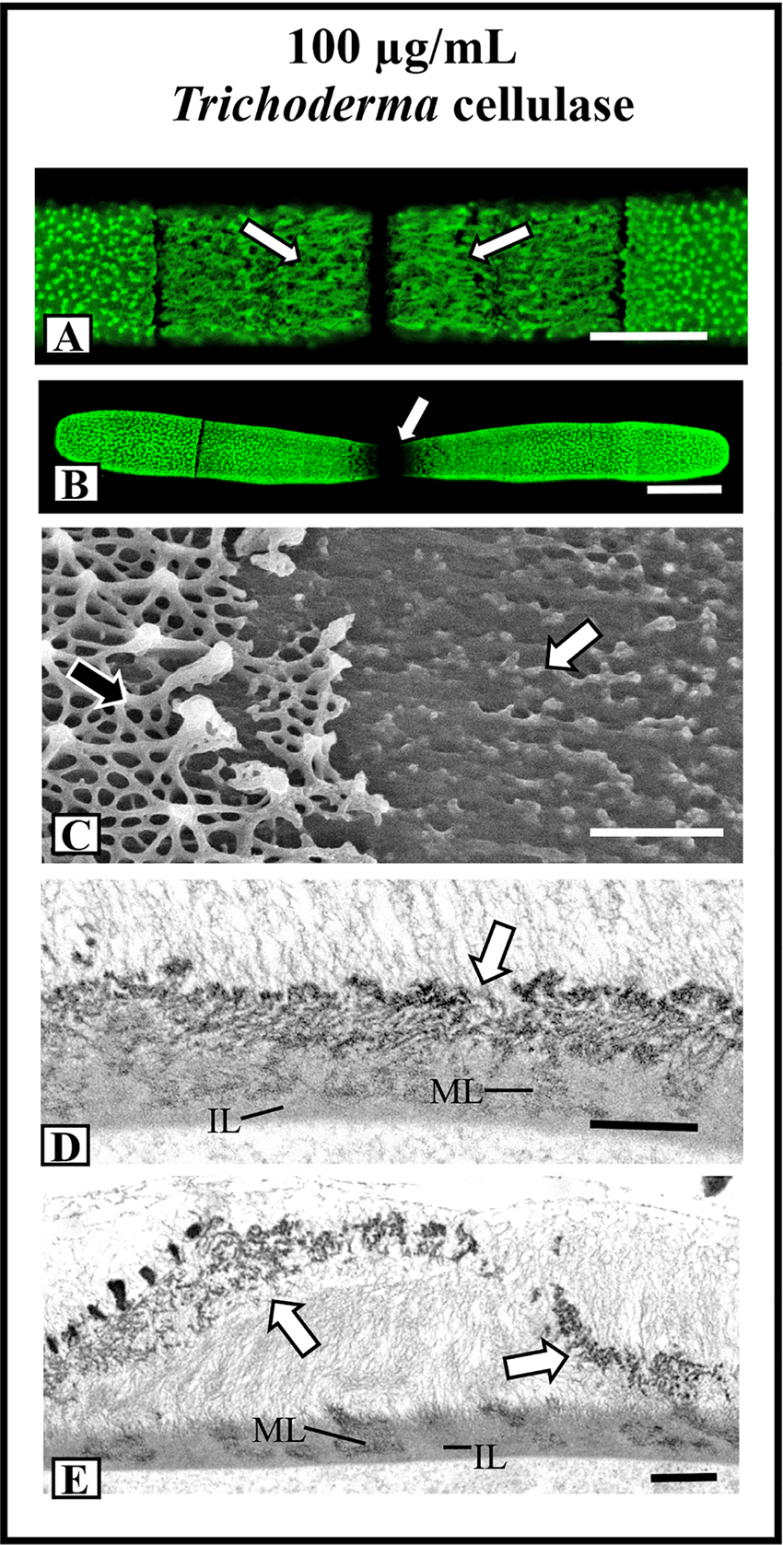
Effects of cellulase treatments on lattice architecture. **(A)** After 12 h of incubation in 100 µg/ml *Trichoderma* cellulose, the lattice transforms into elongate fibers (arrows). OGA7-13^488^ label, confocal laser scanning microscopy (CLSM) image. Bar, 8 µm. **(B)** After 36 h of treatment in cellulose, the cell shape transforms whereby the isthmus narrow and no lattice is apparent (arrow). OGA7-13^488^ label, CLSM image Bar, 17 µm. **(C)** Field emission scanning electron microscopy (FESEM) image of the cell wall surface of a cell treated with cellulose for 36 h. Note the removal of most of the lattice (white arrow). The pre-existing lattice is also observable (black arrow). Bar, 1 µm. **(D)** Transmission electron miscroscopy (TEM) image of the wall of a cell treated with cellulase for 24 h. Note that the lattice is not solid (arrow) but consists of punctate components. The medial (ML) and inner (IL) layer are observable. Bar. 750 nm. **(E)** TEM image of the lattice remnant peeling off the cell wall (arrows) in a cell treated with cellulose for 36 h. The medial (ML) and inner (IL) layer are observable. Bar, 900 nm.

Significant changes to the lattice were observed when cells were incubated in medium containing 100 µg/ml *Trichoderma* cellulase. After 12 h, the lattice at the isthmus transformed into a series of elongated fibers (**Figure 8A**) and after 36 h, the lattice was not seen after labeling withOGA7-13488 (**Figure 8B**). FESEM imaging confirmed the loss of most of the lattice components (**Figure 8C**). TEM imaging showed that the lattice components were no longer solid and consisted of an aggregation of fibers (**Figure 8D**). After 36 h of treatment, the altered lattice appeared to peel off the inner wall layers (**Figure 8E**).

Recovery experiments followed by labeling with OG7-13^488^ are presented in **Supplementary Figure 4**. In all recovery cultures, the HG lattice was produced in the typical isthmus-centered process.

## Discussion

### The Pectin Architecture of the Cell Wall

The cell wall of *Penium* *margaritaceum* is distinguished by an outer layer consisting of Ca^2+^-complexed HG that manifests in a punctate, or “warty” appearance on the cell surface. This layer consists of a basal network of fibers that cover the wall surface, and from which emerge branches that extend above the network, fuse and form distinct point-like projections. While the periodicity of the projections on the wall surface varies somewhat, they are organized in a regular lattice configuration (Domozych et al., 2014a). In this study we interrogated cells with a variety of specific stress agents and monitored alterations to the lattice using multiple microscopy technologies. Our results show that lattice architecture is very sensitive to external stress, is highly malleable and that removal of the stress agent allows for the construction of a new lattice that is initiated at the isthmus, the site of cell expansion.

The foundation of the lattice is the basal fiber, whose substructure consists of large numbers of 5-6 nm wide fibrils that are tightly packed into elongate aggregates. These fibrils are visible using TEM after partial EDTA extraction, i.e., a process that most likely removes complexed Ca^2+^ from the lattice and unmasks the fibrils. The presence of HG fibrils in *Penium*’s wall lattice supports a recent finding that HG forms nanofilaments in *Arabidopsis* pavement cell walls (Haas et al., 2020). The first appearance of basal fibers during cell wall expansion is at the edges of the narrow isthmus, where they arise from the underlying wall layers. The center of the isthmus is devoid of these fibers and does not label with OGA7-13^488^ or JIM5, i.e., probes that recognize low esterified HG. However, the isthmus labels with the RGI-specific mAb, INRA-RU1, and the high esterified HG-specific mAb, JIM7 (see also Domozych et al., 2014a). From these observations, we purport that after the formation of the initial cellulosic wall layer at the isthmus and insertion of RGI, high-esterified HG is incorporated into the wall and either assembles into 5–6 nm fibrils here, or in the vesicles carrying it to the cell surface. It is then deposited onto the wall surface. *High esterified* HG most likely provides a more elastic form of pectin that would more easily move through the inner wall layers. This HG would be less rigid than de-esterified/Ca^2+^-complexed HG and would have less chemical interaction with the cellulose-based inner wall layer (Phyo et al., 2017). After deposition onto the surface, the HG fibrils are de-esterified, and bind with Ca^2+^ to form the basal fiber network of the lattice. De-esterification/Ca^2+^crosslinking of HG is a common chemical modulation in plant cell walls and results in significant changes to the structure and rheology of the pectin (e.g., formation of a stiffened gel) and cell wall in general (Basak and Bandyopadhyay, 2014; Hocq et al., 2017). One might then postulate that the extensive calcified lattice of *Penium* is critical to the maintenance of wall rigidity and in turn, the cylindrical cell shape. However, in our experiments, where the lattice is essentially removed (e.g., full EDTA extraction), cell shape is not compromised. This indicates that the inner wall layers containing cellulose microfibrils provide the foundation for maintenance of cell shape.

EDS analysis of the cell wall surface showed that Ca^2+^ levels increase from the fiber-less isthmus to the initial basal fiber network, to the projections of the mature lattice of the mid semi-cell loci and finally to the polar zones. This sequential increase in Ca^2+^-binding from new to mature lattice zones may be indicative of the period of time that is required during cell expansion for free Ca^2+^ to bind to all available COO- groups on GalA residues exposed during de-esterification. For example, de-esterification/Ca^2+^-binding most likely causes structural modulations to the HG fibril aggregates. These changes could sequentially reveal more –COO- that would then bind to Ca^2+^ during cell expansion. The HG-Ca^2+^-binding would therefore peak at the most mature area of the lattice, the polar zones, and lesser amounts of Ca^2+^ would be found in newly formed lattice and the isthmus. One might also hypothesize that if the de-esterification process is artificially enhanced, greater amounts of –COO- would be exposed and a more rapid and significant Ca^2+^ binding would occur. This was noted in this study when cells were grown in medium supplemented with exogenous orange PME, which resulted in an altered and much thickened lattice. Alternatively, the level of Ca^2+^ that is available in the medium for HG-binding during cell expansion may also contribute to the increasing levels of lattice Ca^2+^ in more mature wall zones. The longer the lattice components are present on the cell surface, the longer it is exposed to available Ca^2+^ and, consequently, the higher amounts of bound Ca^2+^ in the lattice. Higher levels of Ca^2+^ in the growth medium would then be expected to enhance this binding dynamic and affect lattice architecture. In this study, incubation of cells in high Ca^2+^ medium resulted in the formation of a notably thickened lattice with tall projections. Further study will be required to determine Ca^2+^-binding dynamics in the wall including potential mechanisms for regulating Ca^2+^ available for binding. For example, does the extensive extracellular polymeric substance (EPS; Domozych et al., 2005), found just outside the *Penium* cell wall, play a role in sequestering Ca^2+^ for subsequent binding in the wall, as has been described for other EPS-producing algae (Decho and Gutierrez, 2017)? Finally, our EDS analysis also demonstrates that the lattice will complex barium, along with calcium. This indicates that while the HG will bind to other cations, Ca^2+^ is required for forming the typical lattice. Further analyses will be required to determine the range of cations that can be complexed by the lattice.

### The Dynamics of Lattice Deposition

The formation of an *organized* network of basal fibers on the cell wall surface at the isthmus strongly suggests the presence of an *organized* release mechanism for the constituent HG fibrils to pass through the inner wall layer at this zone. One possibility is that the underlying inner layer of the wall has regularly spaced zones that allow for the controlled passage of the HG through the wall to the surface. The size and shape of these zones would regulate the amount of HG fibrils passing through the wall and also, the size/shape of the fibrillar aggregates that ultimately become the lattice fibers. The HG fibrils would be compressed to form an aggregate that are excluded onto the wall surface and whose size is similar to the diameter of the future basal fiber. Continued secretion of these HG fibrillar aggregates onto the narrow isthmus surface would force adjacent fibrillar aggregates to make contact and fuse. Concurrent de-esterification/Ca^2+^ complexing would then rigidify and stabilize the branched meshwork of basal fibers. Likewise, continued secretion of HG would force some of the HG fibrillar aggregates to extend or branch outward from the wall surface. As these extensions grow, they would fuse with other emerging extensions and de-esterification/Ca^2+^-complexing would then create the outer projections of the lattice. It is also possible that upon demethylation, the HG fibrils swell (Zhang and Zhang, 2020; Haas et al., 2020) and these structural changes contribute to basal fiber and the branched lattice formation.

Is there evidence for specialized zones in the inner layer of the cell wall through which the HG is released? First, JIM7 labeling and CLSM imaging revealed a series of tightly packed fluorescent points that are organized in a narrow band at the isthmus. These points represent sites of high esterified HG secretion onto the wall surface and provide structural evidence for a network of closely-spaced zones through which HG is transported. Second, in FESEM and TEM imaging of both the outer and inner surfaces of the isthmus cell wall, no distinct channels (e.g., pores) are evident. However, in this and a previous study (Domozych et al., 2014a), distinct and regular- interruptions do exist in the inner cellulosic wall layer, i.e., the medial cell wall layer. The medial layer consists of branched, tube-like zones that penetrate the inner cellulosic wall layer and open both to the inner and outer wall surfaces. These zones contain aggregates of thin, electron dense fibrils and label with mAbs that recognize both RGI and HG epitopes. Additionally, HG fibrils extend inward from the HG lattice and connect with the medial layer.

**Figure 9:**
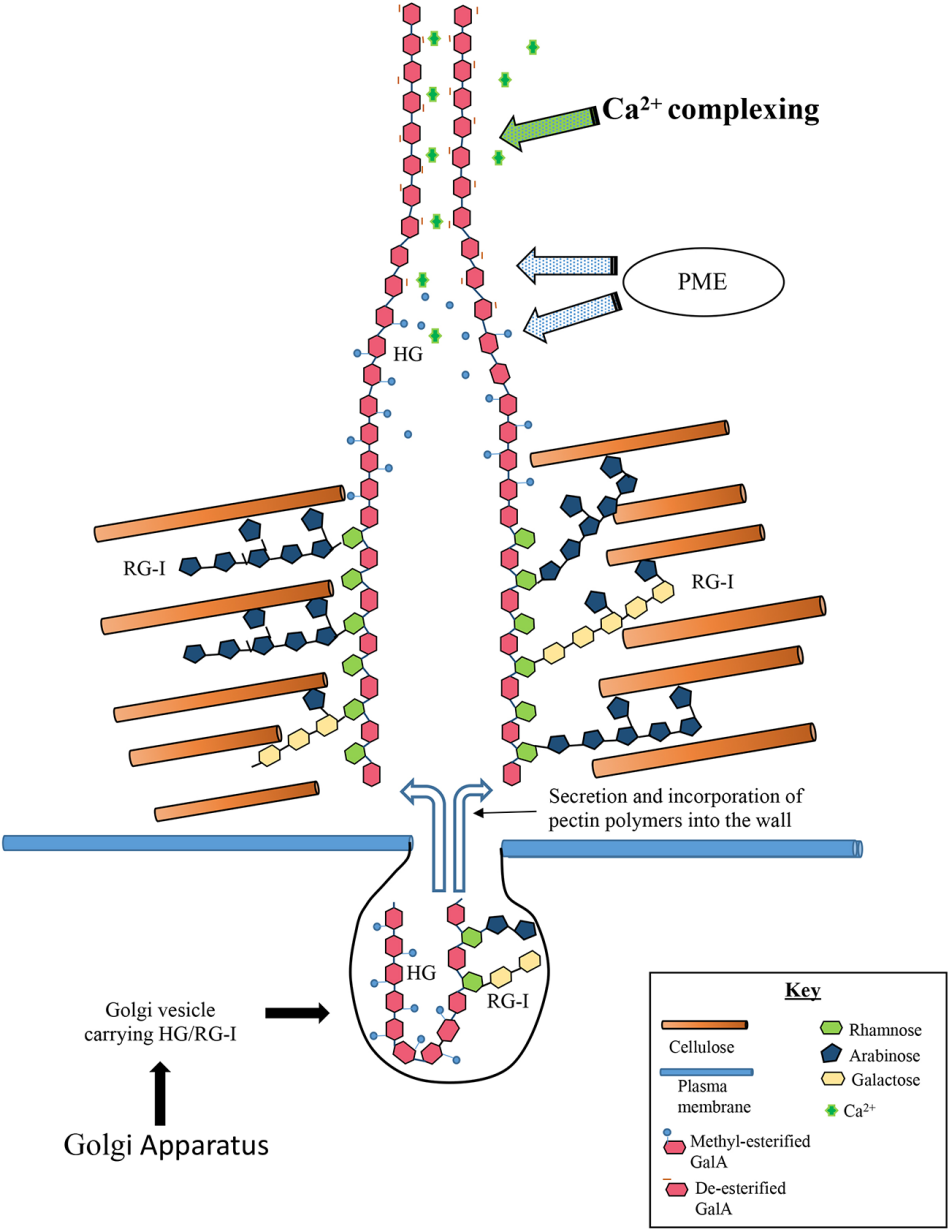
Model of formation of basal fibers. The steps involved in deposition of the homogalacturonan (HG) include: (1) Golgi derived vesicles carry high-esterified HG and rhamnogalacturonan I (RGI), to the isthmus. (2) The HG is secreted into the inner wall layers whereby it enters special zones that allow its passage to the wall surface zones. (3) The RGI component of the pectin associates with surrounding cellulose microfibrils and anchors the HG to the wall. (4) The high-esterified chains of the HG component of the pectin moves through the wall through the specialized zones and deposits onto the wall surface. (5) PME de-esterifies the HG and subsequent Ca^2+^-complexing creates the basal fiber.

We propose a working model of the *Penium* pectin deposition machinery (**Figure 9**). High esterified-HG and RGI are processed by the Golgi apparatus and sent *via* secretory vesicles to the isthmus zone. The vesicles fuse with the plasma membrane and the pectin is secreted into the cellulose layer that is initially made at the isthmus. Distinct structural zones are created whereby the RGI associates with the surrounding cellulose microfibrils. High-esterified HG (fibrillar aggregates) passes through these zones and migrates to the wall surface. Here, PME de-esterifies the HG and subsequent Ca^2+^- complexing creates the basal fibers of the lattice. Our TEM imaging and immunogold labeling lends support to this model by showing that the cellulosic inner layer is produced first and is followed by the deposition of RGI to form the medial layer. This creates a cellulose-RGI infrastructure that is necessary before HG is added to the wall surface. The molecular changes to wall architecture *via* RGI-cellulose interactions that would create the special HG secretion pathways through the wall are not known, but this putative RGI-cellulose association is congruent with recent research demonstrating the affinity of arabinan and galactan side chains of RGI for cellulose microfibrils (Cosgrove, 2014; Wang and Hong, 2016). Our model will require future testing and testing in order to address key questions: Do HG and RGI form a *super* pectin polymer complex? After synthesis in the Golgi apparatus, are they packaged together in the same vesicle or in different vesicles, and is there temporal control of specific vesicle transport to the isthmus? Do the HG fibrils structurally change upon removal of methyl-ester groups like that demonstrated in cotyledons (Haas et al., 2020)? How do HG fibrils organize in inner wall layers and interact with RGI? What mechanism provides the force required to push the HG fibrils through the wall? How is RGI anchored to cellulose? The regular spacing of the lattice projections also strongly supports a highly organized and coordinated, spatiotemporally-controlled HG secretion mechanism. That is, the lattice is a product of HG being secreted at the expanding isthmus at a controlled rate onto a narrow/small surface, and this process is coordinated with de-esterification and Ca^2+^-binding. Furthermore, HG lattice formation is dependent on the prior formation of the cellulosic inner layer. How then is cellulose microfibril synthesis at the isthmus interwoven with pectin secretion?

### Lattice Re-Construction During Experimental Recovery Periods

During recovery periods for virtually all of our experiments, regeneration of a new lattice occurred at the isthmus zone in a manner identical to lattice formation in untreated cells. This highly focused deposition demonstrates that the cortical cytoplasm and plasma membrane of the isthmus contain the necessary subcellular machinery and signaling apparatus for the focused deposition of pectin precursors and their subsequent assembly into lattice. The isthmus-based recovery is best exemplified in our experiment with full EDTA extraction. This treatment removed all, or most, of the lattice and HG present in the wall but did not extract large amounts, if any, of the RGI. However, partial EDTA extraction did not remove all of the lattice components or HG or the RGI from the wall. Interestingly, lattice reformation during recovery from partial extraction occurred not only at the isthmus but also at non-isthmus zones where lattice components and HG in the medial layer remain. These observations indicate that *Penium*, in addition to its isthmus-based lattice deposition apparatus, possesses some type of lattice-“repair” mechanism that exists at non-isthmus regions. We posit that if the foundational HG components, i.e., lattice remnants on the wall surface and HG in the underlying wall layers, remain in regions outside the isthmus, then a lattice repair mechanism will activate and produce new lattice components at these zones. It is also important to note that this lattice repair only yields patches or bands of new lattice components and not large areas of lattice. The purpose of this partial repair remains unresolved, but could be important in producing sufficient amounts of lattice at specific regions of the cell surface that are required for adhesion. Overall though, the isthmus-based, lattice deposition machinery produces a complete lattice during recovery and demonstrates its primacy in the wall developmental apparatus. We also observed that this isthmus-based lattice deposition is the only means of lattice regeneration during recovery in all other experimental treatments and that non-isthmus repair was not present. This might indicate that only partial EDTA extraction leaves the requisite lattice components that activate the repair mechanism.

The existence of a lattice deposition apparatus at non-isthmus regions is also supported by the formation of lateral bands that are found throughout the cell wall surface. These narrow, lattice-free bands are flanked on one edge by a lattice with thickened projections and on the other edge by a lattice with smaller projections. We have interpreted these bands as representing possible expansion oscillation zones where lattice deposition and cell expansion turns on/off during periods of the cell cycle. For example, cell wall expansion/lattice formation turns off in the dark but re-starts in the light and these bands are simply manifestations of these changes in deposition activity. Clearly though, a lattice deposition mechanism must be present at these zones when lattice deposition resumes. The subcellular components and the inclusive signaling networks that identify the specific sites for this type of lattice deposition mechanism will need to be resolved in future studies.

### Osmotic Conditions Affect Lattice Formation

Primary cell wall expansion in plants is a process defined by two major interacting forces, turgor-driven hydrostatic pressure and the tensile resistance of the cell wall to this pressure (Kroeger et al., 2011; Ali and Traas, 2016; Bidhendi et al., 2019). Pectins have also been identified as key polymers whose chemical modulations create localized loosening of wall architecture (Peaucelle et al., 2011; Peaucelle et al., 2012; Haas et al., 2020). Turgor pressure then causes plastic deformation of the wall at these regions, which ultimately leads to cell wall and expansion (Altartouri et al., 2019; Zhao et al., 2019). In our study, pectin architecture was notably disturbed when turgor was reduced by growing cells in hyperosmotic medium (225 mM sorbitol). During this treatment, cell expansion occurred but cytokinesis stopped. This leads to a multinucleate filamentous phenotype. Isthmus-like regions separate cell “units” in the filament and it is here that highly irregular and dispersed patches of altered fibers replace the typical lattice. This structural alteration may be a product of a hyperosmosis-generated asynchrony of cell expansion with lattice deposition. For example, the decrease in turgor may alter both HG delivery of Golgi-derived vesicles to the cell surface and their fusion with the plasma membrane at the isthmus zone during cell expansion. The appearance of large membrane-containing inclusions found between the plasma membrane and cell wall during hyperosmotic treatment would support the possibility of major alterations to the cell secretory machinery. Additionally, hyperosmosis may directly affect the plasma membrane and consequently, its role in cell wall development. Recent studies have shown that hyperosmotic conditions directly alter the plasma membrane, both its tension and its interactions with the cell wall (Nakayama et al., 2012), including alterations to plasma membrane proteins that regulate membrane trafficking, including the balance of exocytosis-endocytosis.

### The Effects of Agents That Disrupt Microtubule and Microfilament Dynamics

The network of cortical microtubules and actin microfilament bundles at the isthmus of *Penium* (Ochs et al., 2014) most likely plays a key role during cell expansion and cell wall development. In this study, we incubated cells in media containing the cytoskeleton-perturbation agents, APM and CB. APM is an agent that depolymerizes microtubules in plant cells (Falconer and Seagull, 1987) and application to *Penium* caused major changes to the lattice. First, APM-treatment resulted in significant swelling at the isthmus. This agent most likely perturbs the cortical microtubule band at the isthmus that, in turn, alters the cellulose synthesis machinery, weakening the inner wall layer infrastructure. This lead to decreased localized resistance to turgor pressure and subsequent swelling at the isthmus. This is similar to what was noted in Domozych et al. (2014b) where *Penium* was treated with the microtubule perturbation agent, oryzalin. During APM treatment, lattice architecture at the isthmus swelling was transformed into a highly irregular array of both patches of the basal fiber network and an irregular array of highly thickened fibers. This alteration was most likely due to the HG deposition mechanism being out of synchrony with the rapid expansion of the cell and cell wall surface (i.e., producing the lattice on a rapidly expanding sphere versus a precisely-sized, narrow cylinder). Likewise, the changes to the underlying cellulosic layer during treatment very likely alter the inner architectural platform required for the production of the lattice. Further work will be needed to determine if alterations to the inner wall infrastructure affect how the HG fibrils are deposited on the surface.

CB is an agent that affects actin filament formation and elongation and stops cytoplasmic streaming without affecting actin bundle structure (Foissner and Wasteneys, 2007). In *Penium*, CB treatment results in the formation of an elongate filamentous phenotype consisting of incompletely-divided cell units. However, disruption of the lattice is minimal. These results indicate that the cell’s peripheral actin network that is affected by CB has little influence on the production of the lattice or cell/cell wall expansion. Yet, CB-induced perturbation has a distinct effect on cytokinesis, i.e., causing incomplete cell division. Actin filaments have been shown to be critical in cell plate formation/cytokinesis in plants (Maeda et al., 2020) and most likely have an important role in cell division in *Penium*. Future studies will be required to dissect the different roles of actin subpopulations in *Penium*.

### The HG Lattice Is Sensitive to Exogenous Pectin Esterases

Pectins are modified by a wide range of enzymes during biosynthesis, secretion and modulation after deposition into the cell wall (Sénéchal et al., 2014). In this study we interrogated *Penium* with various exogenous pectin-specific enzymes and monitored their effects on lattice production.

PME removes the methyl group from C-6 of the GalA residues of HG that consequently exposes free carboxyl groups, often in a block-wise fashion. This then allows for Ca^2+^-crosslinking of adjacent HG chains (Sénéchal et al., 2014) or a swelling of the HG nanofibrils (Haas et al., 2020) that results in structural modulations of the HG. PME-based activity is therefore critical for control of cell and cell wall expansion, cell-cell adhesion, tissue remodeling and defense against certain pathogens (Willats et al., 2001; Jarvis et al., 2003; Levesque-Tremblay et al., 2015). Incubation of *Penium* in exogenous *plant* (i.e., orange) PME resulted in a lattice containing a limited number of the branched basal fibers and no projections. Rather, the lattice comprised a series of elongate and highly thickened fibers (see also Domozych et al., 2014a). The exogenous *plant*-derived PME most likely enhanced the de-esterification of large blocks of -COO- and complexation of Ca^2+^. This hyperaccumulation of Ca^2+^ resulted in rapid thickening of the basal fibers and did not allow for the formation of the typical projections. We also incubated cells in *bacterial* PME, an enzyme that also de-esterifies HG but most often in a random non-block-wise fashion. Here, a very different transformation to the lattice occurred. Highly flattened basal components, most likely alterations of the basal fiber network, covered the wall surface from which small point-like projections occasionally emerged. This may indicate that non-blockwise de-esterification targets and alters HG architecture during early deposition stages (i.e. during passage through the wall or initial deposition on the wall surface), resulting in the inability of the emerging HG fibrillar aggregates to form the basal fibers. Clearly, the spatial targeting of enzyme-based de-esterification and subsequent Ca^2+^ complexing plays a major role in the manifestation of the architectural design of the lattice in *Penium*.

Epigallocatechin is a phenolic compound that has been shown to be a putative inhibitor of PME activity in plants (Lewis et al., 2008). Upon incubation of *Penium* in medium containing epigallocatechin, lattice formation was notably disrupted. The basal fiber network transformed into a highly flattened and irregularly branched network of fibers with no projections. It is possible that epigallocatechin affects the PME activity of *Penium* by reducing de-esterification or changing the de-esterification pattern from a blockwise to a more random fashion, which leads to significant alterations in the basal lattice network. However, it is also possible that a phenolic compound like epigallocatechin may directly interact with the HG (Mercado-Mercado et al., 2020) and change its architecture.

We also incubated *Penium* in medium containing PAE. This enzyme hydrolyzes acetyl-ester bonds at O2 and/or O3 on GalA residues of either HG or RG-1 (Sénéchal et al., 2014). PAE has been shown to affect cell wall viscosity and also impair the formation of Ca^2+^-crosslinking between HG chains (Renard and Jarvis, 1999; Orfila et al., 2012). In *Penium*, PAE treatment transformed the lattice into a series of small patches of basal fibers with irregular thickened extensions. Though nothing is currently known about acetyl esters in *Penium* pectin, the observed PAE-induced changes show that acetyl-esterification most likely plays some role in the deposition mechanism and the architecture of the lattice.

### RG-1 Affecting Enzymes Cause Subtle Changes in HG Lattice Formation

RGI has been identified in the medial layer of the *Penium* cell wall where it is co-located with HG fibrils extending inward from the lattice. To determine the role of RGI in the formation of the lattice, cells were interrogated with enzymes that target either the backbone and or side chains of RGI. Rhamnogalacturonan hydrolase, or RGI hydrolase, is an endohydrolase that cleaves the α4)-α-d-GalA-(1α2)-α-l-Rha glycosidic linkages, while rhamnogalacturonan lyase B, or RGI lyase, is an endolyase that cleaves α2)-α-l-Rha-(1α4)-α-d-GalA glycosidic linkages (Azadi et al., 1995) in the RGI backbone. When cells are incubated in RGI hydrolase or RGl lyase, the major alteration to the lattice is the formation of an incomplete basal fiber network. That is, the network is produced in a patch-like manner and no projections emerge from them. INRA-RU1 labeling of treated cells confirmed that RGI is structurally altered by treatment with these two RGI-targeted enzymes. Likewise, TEM imaging showed that the outer region of the RGI-containing medial layer is disrupted during enzyme treatment. All of these observations indicate that these two enzymes that target the RGI backbone alter the structural integrity of RGI and/or its connection to HG in the medial wall layer. This may then disrupt the normal passage of HG fibrils to the wall surface during expansion and consequently result in the incomplete formation of the basal fiber network and projections.

We also applied enzymes that target the side branches of RGI including the arabinanase, E-EARAB (an enzyme that affects endohydrolysis of (1→5)-α-l-Ara), and alpha-L-rhamnoside r, rhamnohydrolase, E-RHAMS, (an enzyme that affects exohydrolysis of (1→5)-α-l-Rha). Both treatments had minor effects, which included a decrease in branching in the basal fiber network and the absence of projections. These results are similar to what is observed with treatments with RGI hydrolase and RGI lyase and further indicate a significant role of RGI in the formation of the lattice foundation, i.e., the basal fiber network. However, arabinogalactan protein (AGP) has been found in the cell wall of *Penium* (Palacio-Lopez et al., 2019) and it is possible that E-EARAB and E-RHAMS affect these components. AGP has been found to interact with wall polysaccharides including pectins (Tan et al., 2013) and its enzyme-induced perturbation in Penium may be responsible for causing structural changes to the lattice

### Does Cellulose in the Wall Architecture Influence the Formation of the Lattice?

Prior to the formation of the HG lattice during cell expansion, the cellulose inner wall layer must be produced at the isthmus. This cellulose microfibril infrastructure forms the structural platform for the insertion of RGI and the formation of the medial layer and subsequently, the deposition of HG to form the lattice. These suppositions are based on various microscopy-based observations made here and in previous studies (Domozych et al., 2007; Domozych et al., 2014a). To determine the role of cellulose in lattice formation, we incubated cells in *Trichoderma* cellulase. After an initial transformation of the HG lattice into aggregates of fibers, longer treatment results in a severe reduction or absence of lattice components. TEM analysis revealed that the lattice components were no longer solid but consisted of packed fibers that ultimately peeled off the wall. These observations strongly suggest that the cellulose infrastructure of the wall directly affects the formation of the lattice. It may be that the cellulase treatment causes a collapse of the medial layer channels that then directly affect the number and arrangement of HG fibrils allowed onto the surface. Likewise, the peeling of the lattice from the wall indicates that alteration of the cellulose infrastructure affects the attachment efficacy of the HG to the inner wall layers. These putative cellulose-HG interactions in *Penium* add to the growing evidence of a close association between pectin and cellulose in plant primary cell walls and their importance in wall expansion (Cosgrove, 2014; Bidhendi and Geitmann, 2016; Altartouri et al., 2019).

## Conclusion

The pectin lattice of *Penium* is a unique cell wall component that is highly sensitive to external agents that extract the lattice, create hyperosmotic conditions, perturb the cytoskeleton, and enzymes that target different pectin components and cellulose. This highly malleable lattice made of fibers consisting of 5–6 nm fibrils provides a surface topography for the cell that structurally conforms in response to different external and internal stress agents. As important, the lattice reforms upon recovery from treatments with stress agents. In a previous study (Domozych et al., 2014a), it was posited that the pectin lattice of *Penium* might be critical for adhesion, similar to the role of pectin in the middle lamella of multicellular plants. *Penium* though may use its pectin lattice for another adhesion-based phenomenon. Like many taxa of the Zygnematophyceae, *Penium* adheres rapidly and firmly to a variety of surfaces as a necessary first step in becoming sessile and initiating the formation of a biofilm complex. Is it possible that the ability of the lattice to structurally change in response to external prompts is important for conforming to the structural parameters of a particular substrate? This might afford a more stable adhesive interaction between the cell and the substrate. Further work will be needed to examine this possibility. The recent sequencing of *Penium margaritaceum* genome revealed remarkably large families of genes that contribute to pectin biosynthesis and degradation (Jiao et al., 2020), and this information will be valuable in elucidating the enzymatic basis of pectin network formation and modification during cell expansion and under stress conditions. This in turn will allow for a more comprehensive understanding of the subcellular and molecular mechanisms involved in cell wall production of basal streptophytes like *Penium* and provide important insight into cell wall features that may have been key in the transition of aquatic-to-terrestrial habitats for ancient green plants.

## Data Availability Statement

The raw data supporting the conclusions of this article will be made available by the authors, without undue reservation.

## Author Contributions

DD and JR designed research. KP-L, LS, RR, and EK performed research. KP-L, IS, JR, and DD wrote the manuscript. All authors contributed to the article and approved the submitted version.

## Conflict of Interest

The authors declare that the research was conducted in the absence of any commercial or financial relationships that could be construed as a potential conflict of interest.

## Supplementary Material

Supplementary Figure 1 Fine structure of *Penium*’s pectin lattice. **(A)** FESEM overview of cell wall isolated by freeze shattering highlighting the lattice on the wall surface (rectangle). Bar, 3 mm. **(B)** Magnified FESEM image of the HG lattice. Note the basal fiber network (black arrows), the branches that emerge outward (white arrows) from basal meshwork and the projections (stippled arrows) that form from fusion of the branches. Bar, 450 nm. **(C)** FESEM image of polar zone surface showing thicker and more closely spaced projections (arrows). Bar, 2 µm. **(D)** TEM image of HG lattice partially extracted with EDTA (25 mM EDTA, 10 min). Note that the fibers that make up the lattice consist of tightly clustered aggregates of thin fibrils. The basal network (black arrows) and projection (white arrow) are clearly distinguished. Bar. 210 nm. **(E)** TEM image of the fibrils, packed in rough parallel alignments (arrows), that make up the lattice fibers. Bar. 50 nm. **(F)** TEM image of the regular cell wall revealing the outer layer (OL) containing the lattice and the inner layer (IL) containing cellulose microfibrils. The medial layer (ML) forms channels that extend from the inner surface of the wall to the lattice. Bar. 1 µm. (**G**) TEM image showing fibrils from the lattice (arrows) extending inward in the cell wall. Bar. 210 nm. **(H)** Immunogold labeling of the wall with JIM5 (specificity: Homogalacturonan, HG, with low degree of methyl-esterification; Clausen et al., 2003) showing that both the outer layer (OL) and medial layer (ML) label with this mAB. Also, note the branched channel-like network of the medial layer in the inner cellulosic layer (IL). Bar, 200 nm. **(I)** Co-immunogold labeling of the cell wall with JIM5 and INRA-RU1 (specificity: Rha-(1,4)-GalA-(1,2)-Rha-(1,4)-GalA-(1,2)-Rha-(1,4)-Rha-(1,4)-GalA-(1,2)-Rha-(1,4)-GalA-(1,2)-Rha-(1; Ralet et al., 2010). JIM5 labels (white arrows) the outer (OL) and medial (ML) layers while INRA-RU1 only labels the medial layer (black arrow). Bar, 210 nm.

Supplementary Figure 2 The isthmus. **(A)** TEM image of the isthmus zone. Note the absence of the HG lattice. This zone contains cellulose microfibrils (white arrows) and the beginning of the medial layer (black arrow). Bar 500 nm. **(B)** INRA-RU1 (specificity: Rha-(1,4)-GalA-(1,2)-Rha-(1,4)-GalA-(1,2)-Rha-(1,4)-Rha-(1,4)-GalA-(1,2)-Rha-(1,4)-GalA-(1,2)-Rha-(1; Ralet et al., 2010) labels the cell wall at the isthmus (arrows) Bar 750 nm. **(C)** JIM5 (specificity: Homogalacturonan, HG, with low degree of methyl-esterification; Clausen et al., 2003) labels only the developing lattice (arrows). Bar, 750 nm.

Supplementary Figure 3 CBM-64a and INRA-RU1 specificity: Rha-(1,4)-GalA-(1,2)-Rha-(1,4)-GalA-(1,2)-Rha-(1,4)-Rha-(1,4)-GalA-(1,2)-Rha-(1,4)-GalA-(1,2)-Rha-(1; Ralet et al., 2010) labeling. **(A)** CBM-64a (specificity: A carbohydrate binding module derived from *Spirochaeta thermophile* that binds to crystalline forms of cellulose) labeling of a control cell. The labeled cellulose of the inner layer is present throughout the cell (arrow). CLSM image. Bar, 7 µm. **(B)** CBM-64a labeling of an APM-treated cell. Note the disruption of the cellulose inner layer (arrow) at the swollen isthmus. Bar. 10 µm. (**C)** INRA-RU1-labeled cell wall control cell. Note that this antibody labels the wall (arrows) but does not the lattice. Bar. 17 µm. **(D)** INRA-RU1 labeled cell wall of a cell incubated in RG1-lyase. Note that portions of the wall appear to have been digested away (arrows). Bar. 17µm. **(E)** INRA-RU1 labeled cell wall of a cell incubated in rhamnogalacturonan hydrolase. Note that portions of the wall appear to have been removed (arrow). Bar. 8 µm.

Supplementary Figure 4 Recovery of HG lattice (arrows) at isthmus zone after 48-72 h of recovery after various treatments. **(A)** Bar, 5 µm; **(B)** Bar, 8 µm; **(C)** Bar, 7.5 µm; **(D)** Bar, 4 µm; **(E)** Bar, 6.5 µm; **(F)** Bar, 7.5 µm; **(G)** Bar, 7 µm; **(H)** Bar, 7.5 µm; **(I)** 5.5 µm; **(J)** bar, 8 µm; **(K)** 7.5 µm, **(L)** 7.5 µm. OG7-13^488^ labeling and CLSM imaging.

## References

[B1] Agoda-Tandjawa G. Durand S. Gaillard C. Garnier C. Doublier J. L. (2012). Properties of cellulose/pectins composites: Implication for structural and mechanical properties of cell wall. Carbohydr. Polym. 90, 1081–1091. 10.1016/j.carbpol.2012.06.047 22840043

[B2] Ali O. Traas J. (2016). Force-driven polymerization and turgor-induced wall expansion. Trends Plant Sci. 21, 398–409. 10.1016/j.tplants.2016.01.019 26895732

[B3] Altartouri B. Bidhendi A. J. Tani T. Suzuki J. Conrad C. Chebli Y. (2019). Pectin chemistry and cellulose crystallinity govern pavement cell morphogenesis in a multi-step mechanism. Plant Physiol. 181, 127–141. 10.1104/pp.19.00303 31363005 PMC6716242

[B4] Anderson C. T. Wallace I. S. Somerville C. R. (2012). Metabolic click-labeling with a fucose analog reveals pectin delivery, architecture, and dynamics in *Arabidopsis* cell walls. Proc. Natl. Acad. Sci. U. S. A. 109, 1329–1334. 10.1073/pnas.1120429109 22232683 PMC3268317

[B5] Anderson C. T. (2016). We be jammin’: an update on pectin biosynthesis, trafficking and dynamics. J. Exp. Bot. 67, 495–502. 10.1093/jxb/erv501 26590862

[B6] Azadi P. O’Neill M. A. Bergmann C. Darvill A. G. Albersheim P. (1995). The backbone of the pectic polysaccharide rhamnogalacturonan I is cleaved by an endohydrolase and an endolyase. Glycobiology 5, 783–789. 10.1093/glycob/5.8.783 8720076

[B7] Basak R. Bandyopadhyay R. (2014). Formation and rupture of Ca(2+) induced pectin biopolymer gels. Soft Matter 10, 7225–7233. 10.1039/c4sm00748d.\ 25160564

[B8] Bidhendi A. J. Geitmann A. (2016). Relating the mechanics of the primary plant cell wall to morphogenesis. J. Exp. Bot. 67, 449–461. 10.1093/jxb/erv535 26689854

[B9] Bidhendi A. J. Altartouri B. Gosselin F. P. Geitmann A. (2019). Mechanical stress initiates and sustains the morphogenesis of wavy leaf epidermal cells. Cell Rep. 28, 1237–1250.e6. 10.1016/j.celrep.2019.07.006 31365867

[B10] Biswal A. K. Atmodjo M. A. Li M. Baxter H. L. Yoo C. G. Pu Y. (2018). Sugar release and growth of biofuel crops are improved by downregulation of pectin biosynthesis. Nat. Biotechnol. 36, 249–257. 10.1038/nbt.4067 29431741

[B11] Boyer J. S. (2009). Cell wall biosynthesis and the molecular mechanism of plant enlargement. Funct. Plant Biol. 36, 383–394. 10.1071/FP09048 32688655

[B12] Caffall K. H. Mohnen D. (2009). The structure, function, and biosynthesis of plant cell wall pectic polysaccharides. Carbohydr. Res. 344, 1879–1900. 10.1016/j.carres.2009.05.021 19616198

[B13] Clausen M. H. Willats W. G. T. Knox J. P. (2003). Synthetic methyl hexagalacturonate hapten inhibitors of anti-homogalacturonan monoclonal antibodies LM7, JIM5 and JIM7. Carbohydr. Res. 338:, 1797–1800. 10.1016/S0008-6215(03)00272-6 12892947

[B14] Cosgrove D. J. (2014). Re-constructing our models of cellulose and primary cell wall assembly. Curr. Opin. Plant Biol. 22, 122–131. 10.1016/j.pbi.2014.11.001 25460077 PMC4293254

[B15] Decho A. W. Gutierrez T. (2017). Microbial Extracellular Polymeric Substances (EPSs) in ocean systems. Front. Microbiol. 8, 922. 10.3389/fmicb.2017.00922 28603518 PMC5445292

[B16] Delwiche C. F. Cooper E. D. (2015). The evolutionary origin of a terrestrial flora. Cur. Biol. 25, R899–R910. 10.1016/j.cub.2015.08.029 26439353

[B17] Domozych D. S. Kort S. Benton S. Yu T. (2005). The extracellular polymeric substance of the green alga *Penium margaritaceum* and its role in biofilm formation. Biofilms 2, 1–16. 10.1017/S147905050500181X

[B18] Domozych D. S. Serfis A. Kiemle S. N. Gretz M. R. (2007). The structure and biochemistry of charophycean cell walls: I. Pectins of *Penium margaritaceum* . Protoplasma 230, 99–115. 10.1007/s00709-006-0197-8 17111095

[B19] Domozych D. S. Sørensen I. Popper Z. A. Ochs J. Andreas A. Fangel J. U. (2014a). Pectin metabolism and assembly in the cell wall of the charophyte green alga *Penium margaritaceum* . Plant Physiol. 165, 105–118. 10.1104/pp.114.236257 24652345 PMC4012572

[B20] Domozych D. S. Sørensen I. Sacks C. Brechka H. Andreas A. Fangel J. U. (2014b). Disruption of the microtubule network alters cellulose deposition and causes major changes in pectin distribution in the cell wall of the green alga, *Penium margaritaceum* . J. Exp. Bot. 65, 465–479. 10.1093/jxb/ert390 24285826 PMC3904706

[B21] Domozych D. Popper Z. A. Sorensen I. (2016). Charophytes: Evolutionary giants and emerging model organisms. Front. Plant Sci. 7, 1470. 10.3389/fpls.2016.01470 27777578 PMC5056234

[B22] Eder M. Lütz-Meindl U. (2010). Analyses and localization of pectin-like carbohydrates in cell wall and mucilage of the green alga *Netrium digitus* . Protoplasma 243, 25–38. 10.1007/s00709-009-0040-0 19340523 PMC2892062

[B23] Falconer M. M. Seagull R. W. (1987). Amiprophos-methyl (APM): A rapid, reversible, anti-microtuble agent for plant cell cultures. Protoplasma 136, 118–124. 10.1007/BF01276360

[B24] Foissner I. Wasteneys G. O. (2007). Wide-ranging effects of eight cytochalasins and latrunculin A and B on intracellular motility and actin filament reorganization in characean internodal cells. Plant Cell Physiol. 48, 585–597. 10.1093/pcp/pcm030 17327257

[B25] Francocci F. Bastianelli E. Lionetti V. Ferrari S. De Lorenzo G. Bellincampi D. (2013). Analysis of pectin mutants and natural accessions of Arabidopsis highlights the impact of de-methyl-esterified homogalacturonan on tissue saccharification. Biotechnol. Biofuels 6, 163. 10.1186/1754-6834-6-163 24245704 PMC3843582

[B26] Guo X. Runavot J.-L. Bourot S. Meulewaeter F. Hernandez-Gomez M. Holland C. (2019). Metabolism of polysaccharides in dynamic middle lamellae during cotton fibre development. Planta 249, 1565–1581. 10.1007/s00425-019-03107-4 30737556

[B27] Haas K. T. Wightman R. Myerowitz E. M. Peaucelle A. (2020). Pectin homogalacturonan nanofilament expansion drives morphogenesis in plant epidermal cells. Science 367, 1003–1007. 10.1126/science.aaz5103 32108107 PMC7932746

[B28] Hocq L. Pelloux J. Lefebvre V. (2017). Connecting homogalacturonan-type pectin remodeling to acid growth. Trends Plant Sci. 22, 20–29. 10.1016/j.tplants.2016.10.009 27884541

[B29] Imaizumi T. Szymańska-Chargot M. Pieczywek P. M. Chylińska M. Kozioł A. Ganczarenko D. (2017). Evaluation of pectin nanostructure by atomic force microscopy in blanched carrot. LWT 84, 658–667. 10.1016/j.lwt.2017.06.038

[B30] Jarvis M. C. Briggs S. P. H. Knox J. P. (2003). Intercellular adhesion and cell separation in plants. Plant Cell Environ. 26, 977–989. 10.1046/j.1365-3040.2003.01034.x

[B31] Jiao C. Sørensen I. Sun X. Behar H. Alseekh S. Philippe G. (2020). The Genome of the charophyte alga *Penium margaritaceum* bears footprints of terrestrialization and preludes the evolutionary origins of land plants. Cell 181, 1–15. 10.1016/j.cerll.2020.04.019 32243785

[B32] Kirby A. R. MacDougall A. J. Morris V. J. (2008). Atomic force microscopy of tomato and sugar beet pectin molecules. Carbohyd. Polym. 71, 640–647. 10.1016/j.carbpol.2007.07.014

[B33] Knox J. P. Linstead P. J. King J. Cooper C. Roberts K. (1990). Pectin esterification is spatially regulated both within cell walls and between developing tissues of root apices. Planta 181, 512–521. 10.1007/BF00193004 24196931

[B34] Kroeger J. H. Zerzour R. Geitmann A. (2011). Regulator or Driving Force? The Role of turgor pressure in oscillatory plant cell growth. PloS One 6, e18549. 10.1371/journal.pone.0018549 21541026 PMC3081820

[B35] Levesque-Tremblay G. Pelloux J. Braybrook S. A. Müller K. (2015). Tuning of pectin methylesterification: consequences for cell wall biomechanics and development. Planta 242, 791–811. 10.1007/s00425-015-2358-5 26168980

[B36] Lewis K. C. Selzer T. Shahar C. Udi Y. Tworowski D. Sagi I. (2008). Inhibition of pectin methyl esterase activity by green tea catechins. Phytochemistry 69, 2586–2592. 10.1016/j.phytochem.2008.08.012 18829053

[B37] Maeda K. Sasabe M. Hanamata S. Machida Y. Hasezawa S. Higaki T. (2020). Actin filament disruption alters phragmoplast microtubule dynamics during the initial phase of plant cytokinesis. Plant Cell Physiol. 61, 445–456. 10.1093/pcp/pcaa003 32030404

[B38] Mercado-Mercado G. de la Rosa L. A. Alvarez-Parilla E. (2020). Effect of pectin on interactions among phenolic compounds determined by antioxidant capacity. J. Mol. Struct. 1199, 12967. 10.1016/j.molstruc.2019.126967

[B39] Mohnen D. (2008). Pectin structure and biosynthesis. Curr. Opin. Plant Biol. 11, 266–277. 10.1016/j.pbi.2008.03.006 18486536

[B40] Mravec J. Kračun S. K. Rydahl M. G. Westereng B. Miart F. Clausen M. H. (2014). Tracking developmentally regulated post-synthetic processing of homogalacturonan and chitin using reciprocal oligosaccharide probes. Development 141, 4841–4850. 10.1242/dev.113365 25395456

[B41] Mravec J. Guo X. Hansen A. R. Schückel J. Kračun S. K. Mikkelsen M. D. (2017a). Pea Border Cell Maturation and Release Involve Complex Cell Wall Structural Dynamics. Plant Physiol. 174, 1051–1066. 10.1104/pp.16.00097 28400496 PMC5462005

[B42] Mravec J. Kračun S. K. Rydahl M. G. Westereng B. Pontiggia D. Lorenzo G. D. (2017b). An oligogalacturonide-derived molecular probe demonstrates the dynamics of calcium-mediated pectin complexation in cell walls of tip-growing structures. Plant J. 91, 534–546. 10.1111/tpj.13574 28419587

[B43] Nakayama M. Kaneko Y. Miyazawa Y. Fujii N. Higashitani N. Wada S. (2012). A possible involvement of autophagy in amyloplast degradation in columella cells during hydrotropic response of Arabidopsis roots. Planta 236, 999–1012. 10.1007/s00425-012-1655-5 22532286

[B44] Nichols (1987). “Growth media-freshwater,” in Handbook of phycological methods: culture methods and growth measurements. (New York: Cambridge University Press).

[B45] Ochs J. LaRue T. Tinaz B. Yongue C. Domozych D. S. (2014). The cortical cytoskeletal network and cell-wall dynamics in the unicellular charophycean green alga *Penium margaritaceum* . Ann. Bot. 114, 1237–1249. 10.1093/aob/mcu013 24603606 PMC4195542

[B46] Orfila C. Degan F. D. Jørgensen B. Scheller H. V. Ray P. M. Ulvskov P. (2012). Expression of mung bean pectin acetyl esterase in potato tubers: effect on acetylation of cell wall polymers and tuber mechanical properties. Planta 236, 185–196. 10.1007/s00425-012-1596-z 22293853

[B47] Palacio-Lopez K. Tinaz B. Holzinger A. Domozych D. D. (2019). Arabinogalactan proteins and the extracellular matrix of charophytes: a sticky business. Front. Plant Sci. 10, 447. 10.3389/fpls.2019.00447 31031785 PMC6474363

[B48] Palin R. Geitmann A. (2012). The role of pectin in plant morphogenesis. Biosystems 109, 397–402. 10.1016/j.biosystems.2012.04.006 22554809

[B49] Paniagua C. Posé S. Morris V. J. Kirby A. R. Quesada M. A. Mercado J. A. (2014). Fruit softening and pectin disassembly: an overview of nanostructural pectin modifications assessed by atomic force microscopy. Ann. Bot. 114, 1375–1383. 10.1093/aob/mcu149 25063934 PMC4195560

[B50] Peaucelle A. Braybrook S. A. Le Guillou L. Bron E. Kuhlemeier C. Höfte H. (2011). Pectin-induced changes in cell wall mechanics underlie organ initiation in *Arabidopsis* . Curr. Biol. 21, 1720–1726. 10.1016/j.cub.2011.08.057 21982593

[B51] Peaucelle A. Braybrook S. A. Höfte H. (2012). Cell wall mcechanics and growth in plants: the role of pectins revisited. Fronti. Plant Sci. 3, 121. 10.3389/fpls.2012.00121 PMC3368173 22685449

[B52] Phyo P. Wang T. Xiao C. Anderson C. T. Hong M. (2017). Effects of pectin molecular weight changes on the structure, dynamics, and polysaccharide interactions of primary cell walls of *Arabidopsis thaliana*: insights from solid-state NMR. Biomacromolecules 18, 2937–2950. 10.1021/acs.biomac.7b00888 28783321

[B53] Proseus T. E. Boyer J. S. (2012a). Pectate chemistry links cell expansion to wall deposition in Chara corallina. Plant Signal Behav. 7, 1490–1492. 10.4161/psb.21777 22918500 PMC3548876

[B54] Proseus T. E. Boyer J. S. (2012b). Calcium deprivation disrupts enlargement of Chara corallina cells: further evidence for the calcium pectate cycle. J. Exp. Bot. 63, 3953–3958. 10.1093/jxb/ers089 22442410 PMC3388837

[B55] Ralet M.-C. Tranquet O. Poulain D. Moïse A. Guillon F. (2010). Monoclonal antibodies to rhamnogalacturonan I backbone. Planta 231, 1373–1383. 10.1007/s00425-010-1116-y 20309579

[B56] Renard C. M. G. C. Jarvis M. C. (1999). Acetylation and methylation of homogalacturonans 2: effect on ion-binding properties and conformations. Carbohydr. Polym. 39, 209–216. 10.1016/S0144-8617(99)00015-6

[B57] Rensing S. (2018). Great moments in evolution: the conquest of land by plants. Curr. Opin. Plant Biol.y 42, 49–54. 10.1016/j.pbi.2018.02.006 29525128

[B58] Sénéchal F. Wattier C. Rustérucci C. Pelloux J. (2014). Homogalacturonan-modifying enzymes: structure, expression, and roles in plants. J. Exp. Bot. 65, 5125–5160. 10.1093/jxb/eru272 25056773 PMC4400535

[B59] Sørensen I. Pettolino F. A. Bacic A. Ralph J. Lu F. O’Neill M. A. (2011). The charophycean green algae provide insights into the early origins of plant cell walls. Plant J. 68, 201–211. 10.1111/j.1365-313X.2011.04686.x 21707800

[B60] Tan L. Eberhard S. Pattathil S. Warder C. Glushka J. Yuan C. (2013). An Arabidopsis cell wall proteoglycan consists of pectin and arabinoxylan covalently linked to an arabinogalactan protein. Plant Cell 25, 270–287. 10.1105/tpc.112.107334 23371948 PMC3584541

[B61] Vincken J.-P. Schols H. A. Oomen R. J. F. J. McCann M. C. Ulvskov P. Voragen A. G. J. (2003). If homogalacturonan were a side chain of rhamnogalacturonan i. implications for cell wall architecture. Plant Physiol. 132, 1781–1789. 10.1104/pp.103.022350 12913136 PMC1540329

[B62] Voxeur A. Höfte H. (2016). Cell wall integrity signaling in plants: “To grow or not to grow that’s the question.” Glycobiology 26, 950–960. 10.1093/glycob/cww029 26945038

[B63] Wang T. Hong M. (2016). Solid-state NMR investigations of cellulose structure and interactions with matrix polysaccharides in plant primary cell walls. J. Exp. Bot. 67:, 503–514. 10.1093/jxb/erv416 26355148 PMC6280985

[B64] Wang T. Park Y. B. Cosgrove D. J. Hong M. (2015). Cellulose-Pectin Spatial contacts are inherent to never-dried arabidopsis primary cell walls: evidence from solid-state nuclear magnetic resonance. Plant Physiol. 168, 871–884. 10.1104/pp.15.00665 26036615 PMC4741345

[B65] Wang D. Samsulrizal N. H. Yan C. Allcock N. S. Craigon J. Blanco-Ulate B. (2019). Characterization of CRISPR mutants targeting genes modulating pectin degradation in ripening tomato. Plant Physiol. 179, 544–557. 10.1104/pp.18.01187 30459263 PMC6426429

[B66] Wasteneys G. O. Willingale-Theune J. Menzel D. (1997). Freeze shattering: a simple and effective method for permeabilizing higher plant cell walls. J. Microsc. 188, 51–61. 10.1046/j.1365-2818.1977.2390796.x 9369020

[B67] Willats W. G. Orfila C. Limberg G. Buchholt H. C. van Alebeek G. J. Voragen A. G. (2001). Modulation of the degree and pattern of methyl-esterification of pectic homogalacturonan in plant cell walls. Implications for pectin methyl esterase action, matrix properties, and cell adhesion. J. Biol. Chem. 276, 19404–19413. 10.1074/jbc.M011242200 11278866

[B68] Yapo B. M. Gnakri D. (2015). “Pectic Polysaccharides and Their Functional Properties,” in Polysaccharides: Bioactivity and Biotechnology. Eds. Ramawat K. G. Mérillon J.-M. (Cham: Springer International Publishing), 1729–1749. 10.1007/978-3-319-16298-0_62

[B69] Zhang D. Zhang B. (2020). Pectin Drives Cell Wall Morphogenesis without Turgor Pressure. Trends Plant Sci. 10.1016/j.tplants.2020.05.007 32513584

[B70] Zhao Y. Man Y. Wen J. Guo Y. Lin J. (2019). Advances in imaging plant cell walls. Trends Plant Sci. 24, 867–878. 10.1016/j.tplants.2019.05.009 31257154

