# Supplementary figures and images for: Experimental Manipulation of Pectin Architecture in the Cell Wall of the Unicellular Charophyte, Penium Margaritaceum
Source: Front Plant Sci. 2020 Jul 8;11:1032. doi: 10.3389/fpls.2020.01032 (PMC7360812; doi:10.3389/fpls.2020.01032)

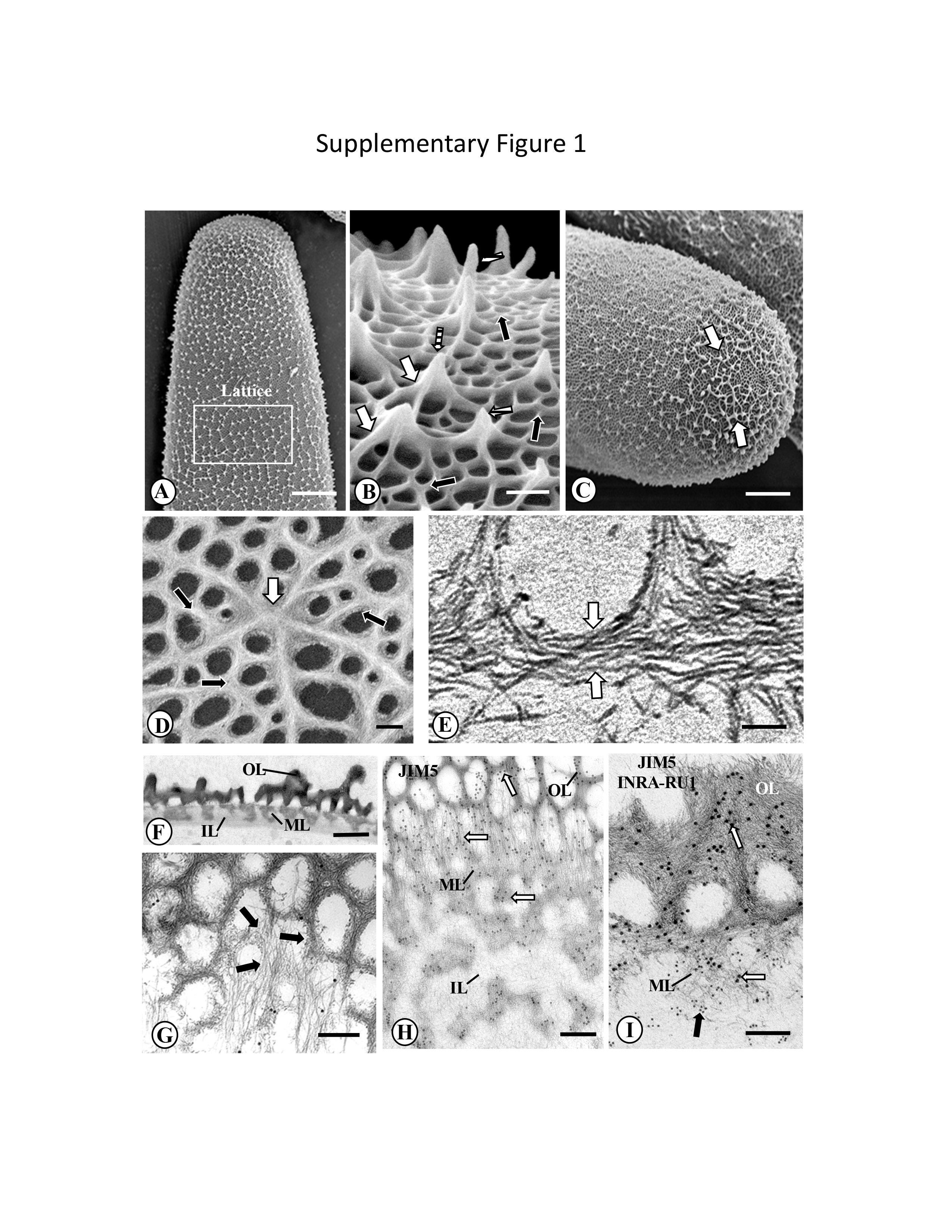

Supplement: Supplementary Figure 1 — Fine structure of Penium’s pectin lattice. (A) FESEM overview of cell wall isolated by freeze shattering highlighting the lattice on the wall surface (rectangle). Bar, 3 mm. (B) Magnified FESEM image of the HG lattice. Note the basal fiber network (black arrows), the branches that emerge outward (white arrows) from basal meshwork and the projections (stippled arrows) that form from fusion of the branches. Bar, 450 nm. (C) FESEM image of polar zone surface showing thicker and more closely spaced projections (arrows). Bar, 2 µm. (D) TEM image of HG lattice partially extracted with EDTA (25 mM EDTA, 10 min). Note that the fibers that make up the lattice consist of tightly clustered aggregates of thin fibrils. The basal network (black arrows) and projection (white arrow) are clearly distinguished. Bar. 210 nm. (E) TEM image of the fibrils, packed in rough parallel alignments (arrows), that make up the lattice fibers. Bar. 50 nm. (F) TEM image of the regular cell wall revealing the outer layer (OL) containing the lattice and the inner layer (IL) containing cellulose microfibrils. The medial layer (ML) forms channels that extend from the inner surface of the wall to the lattice. Bar. 1 µm. (G) TEM image showing fibrils from the lattice (arrows) extending inward in the cell wall. Bar. 210 nm. (H) Immunogold labeling of the wall with JIM5 (specificity: Homogalacturonan, HG, with low degree of methyl-esterification; Clausen et al., 2003) showing that both the outer layer (OL) and medial layer (ML) label with this mAB. Also, note the branched channel-like network of the medial layer in the inner cellulosic layer (IL). Bar, 200 nm. (I) Co-immunogold labeling of the cell wall with JIM5 and INRA-RU1 (specificity: Rha-(1,4)-GalA-(1,2)-Rha-(1,4)-GalA-(1,2)-Rha-(1,4)-Rha-(1,4)-GalA-(1,2)-Rha-(1,4)-GalA-(1,2)-Rha-(1; Ralet et al., 2010). JIM5 labels (white arrows) the outer (OL) and medial (ML) layers while INRA-RU1 only labels the medial layer (black arrow). Bar, 210 nm. [file Image_1.jpeg]

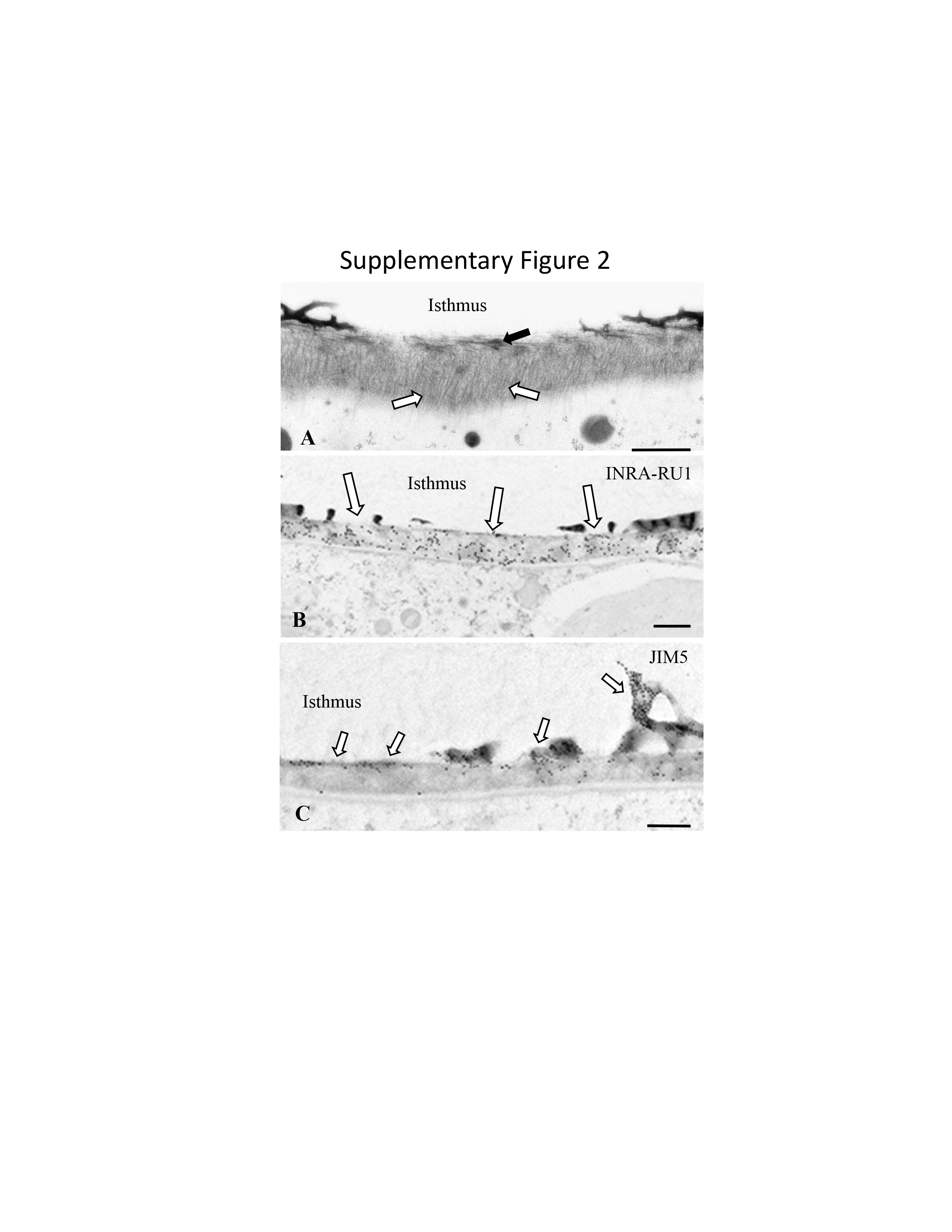

Supplement: Supplementary Figure 2 — The isthmus. (A) TEM image of the isthmus zone. Note the absence of the HG lattice. This zone contains cellulose microfibrils (white arrows) and the beginning of the medial layer (black arrow). Bar 500 nm. (B) INRA-RU1 (specificity: Rha-(1,4)-GalA-(1,2)-Rha-(1,4)-GalA-(1,2)-Rha-(1,4)-Rha-(1,4)-GalA-(1,2)-Rha-(1,4)-GalA-(1,2)-Rha-(1; Ralet et al., 2010) labels the cell wall at the isthmus (arrows) Bar 750 nm. (C) JIM5 (specificity: Homogalacturonan, HG, with low degree of methyl-esterification; Clausen et al., 2003) labels only the developing lattice (arrows). Bar, 750 nm. [file Image_2.jpeg]

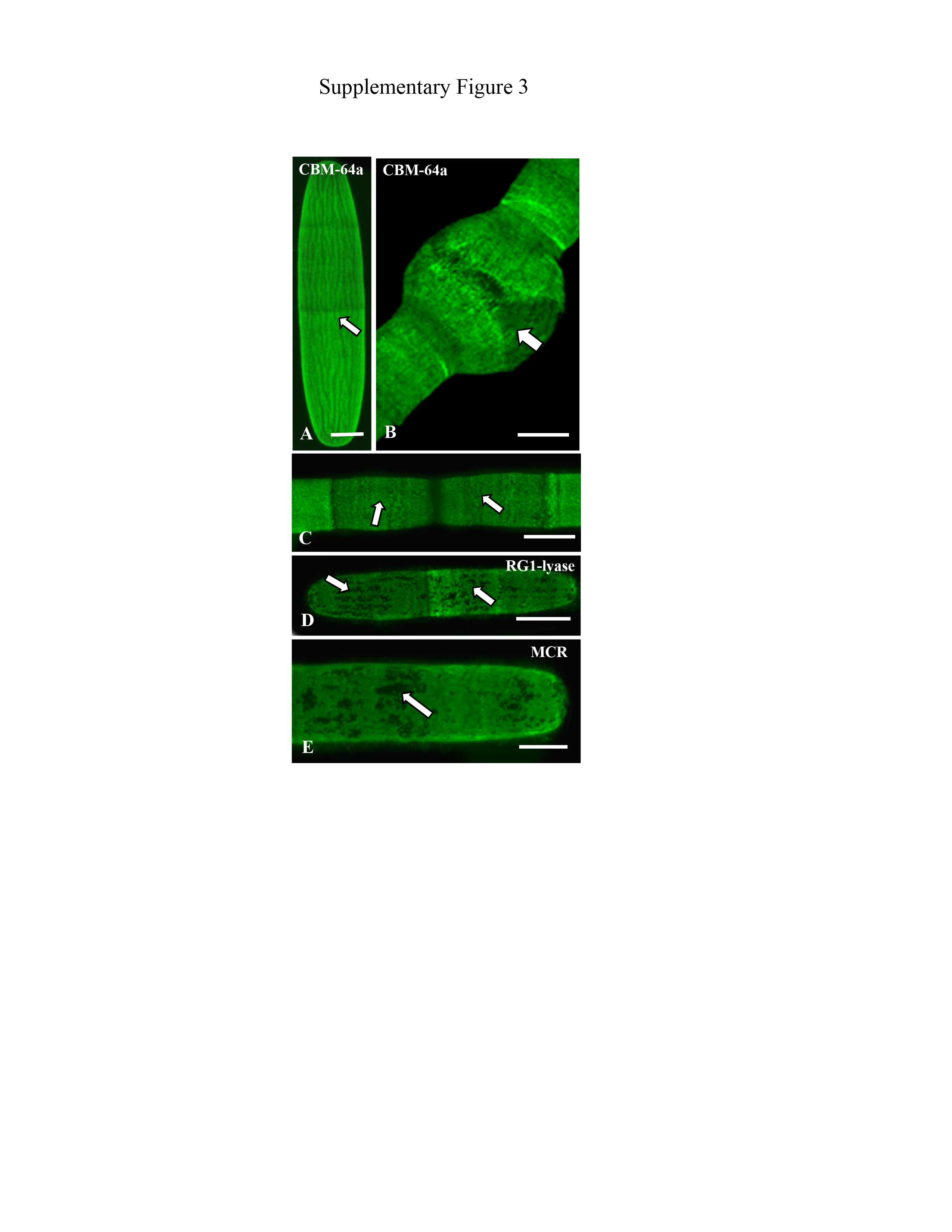

Supplement: Supplementary Figure 3 — CBM-64a and INRA-RU1 specificity: Rha-(1,4)-GalA-(1,2)-Rha-(1,4)-GalA-(1,2)-Rha-(1,4)-Rha-(1,4)-GalA-(1,2)-Rha-(1,4)-GalA-(1,2)-Rha-(1; Ralet et al., 2010) labeling. (A) CBM-64a (specificity: A carbohydrate binding module derived from Spirochaeta thermophile that binds to crystalline forms of cellulose) labeling of a control cell. The labeled cellulose of the inner layer is present throughout the cell (arrow). CLSM image. Bar, 7 µm. (B) CBM-64a labeling of an APM-treated cell. Note the disruption of the cellulose inner layer (arrow) at the swollen isthmus. Bar. 10 µm. (C) INRA-RU1-labeled cell wall control cell. Note that this antibody labels the wall (arrows) but does not the lattice. Bar. 17 µm. (D) INRA-RU1 labeled cell wall of a cell incubated in RG1-lyase. Note that portions of the wall appear to have been digested away (arrows). Bar. 17µm. (E) INRA-RU1 labeled cell wall of a cell incubated in rhamnogalacturonan hydrolase. Note that portions of the wall appear to have been removed (arrow). Bar. 8 µm. [file Image_3.jpeg]

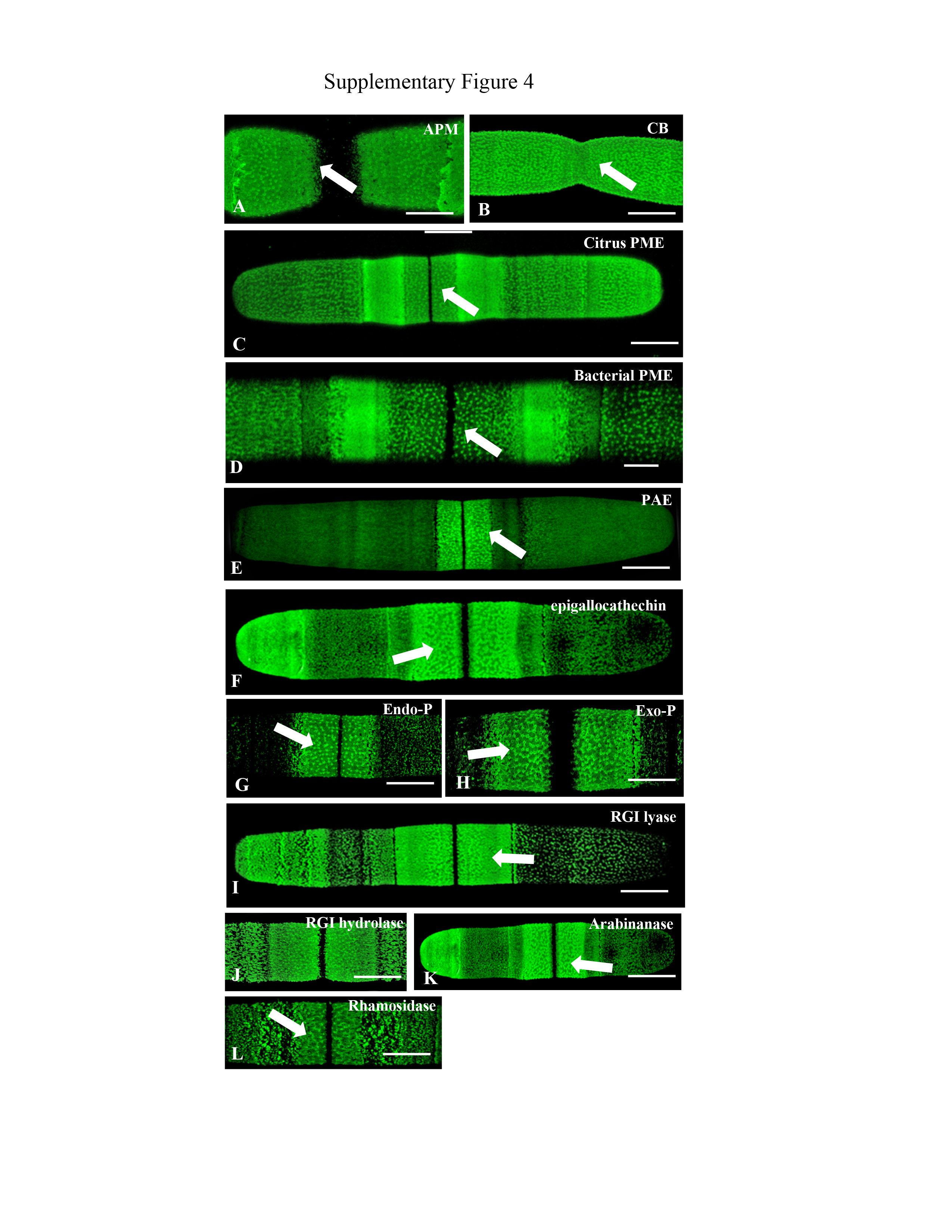

Supplement: Supplementary Figure 4 — Recovery of HG lattice (arrows) at isthmus zone after 48-72 h of recovery after various treatments. (A) Bar, 5 µm; (B) Bar, 8 µm; (C) Bar, 7.5 µm; (D) Bar, 4 µm; (E) Bar, 6.5 µm; (F) Bar, 7.5 µm; (G) Bar, 7 µm; (H) Bar, 7.5 µm; (I) 5.5 µm; (J) bar, 8 µm; (K) 7.5 µm, (L) 7.5 µm. OG7-13488 labeling and CLSM imaging. [file Image_4.jpeg]
